# Impact of climate change on SARS-CoV-2 epidemic in China

**DOI:** 10.1371/journal.pone.0285179

**Published:** 2023-07-27

**Authors:** Zhenyu Yu, Jinnian Wang, Zixuan Tan, Yiyun Luo

**Affiliations:** 1 School of Geography and Remote Sensing, Guangzhou University, Guangdong, China; 2 Innovation Center for Remote Sensing Big Data Intelligent Applications, Guangzhou University, Guangdong, China; Arab Academy for Science Technology and Maritime Transport, EGYPT

## Abstract

The outbreak and prevalence of SARS-CoV-2 have severely affected social security. Physical isolation is an effective control that affects the short-term human-to-human transmission of the epidemic, although weather presents a long-term effect. Understanding the effect of weather on the outbreak allow it to be contained at the earliest possible. China is selected as the study area, and six weather factors that receive the most attention from January 20, 2020 to April 30, 2020 are selected to investigate the correlation between weather and SARS-CoV-2 to provide a theoretical basis for long-term epidemic prevention and control. The results show that (1) the average growth rate (GR) of SARS-CoV-2 in each province is logarithmically distributed with a mean value of 5.15%. The GR of the southeastern region is higher than that of the northwestern region, which is consistent with the Hu Line. (2) The specific humidity, 2-m temperature (T), ultraviolet (UV) radiation, and wind speed (WS) adversely affect the GR. By contrast, the total precipitation (TP) and surface pressure (SP) promote the GR. (3) For every 1 unit increase in UV radiation, the GR decreases by 0.30% in 11 days, and the UV radiation in China is higher than that worldwide (0.92% higher per day). Higher population aggregation and urbanization directly affect the epidemic, and weather is an indirect factor.

## Introduction

In late 2019, the coronavirus was discovered in multiple locations worldwide and began spreading worldwide in 2020 [1, 2]. Coronaviruses are a large family of viruses known to cause severe diseases, such as cold, the Middle East respiratory syndrome (MERS), and the severe acute respiratory syndrome (SARS) [3–5]. On January 12, 2020, the World Health Organization officially named this novel coronavirus, “2019-nCoV”. On February 11, 2020, the International Committee for Virus Taxonomy announced its official name as SARS-CoV-2 [6]. As of May 20, 2020, 215 outbreaks of SARS-CoV-2 have been reported in 215 countries worldwide, with more than 4,895,000 cumulative diagnoses and 324,000 cumulative deaths, and have received significant attention worldwide [7, 8]. The outbreak of SARS-CoV-2 pose a severe risk to social security. Understanding the effects of weather on an outbreak allows the spread of the outbreak to be contained at the earliest possible.

Epidemics involving the human-to-human transmission of coronaviruses have occurred in regions with subtropical monsoon climates in the Northern Hemisphere during the winter and spring [9, 10]. Global warming and extreme weather affect disease outbreaks and transmission [10]. Suitable weather conditions facilitate outbreaks and epidemics. SARS and MERS coronavirus outbreaks in the Coronaviridae family first occurred in regions of subtropical monsoon climates, e.g., SARS in Guangdong Province, China, in 2002 [11]. The Yellow Fever [12], dengue [13], and Zika virus [14] have caused several outbreaks, mainly in the tropical regions of South America, Central America, and Africa, and also in tropical countries in Asia, where the risk varies depending on precipitation, temperature, and rapid urbanization [15, 16]. Recent studies have shown that low temperatures promote the spread of the SARS-CoV-2 infection. Wang et al. [17] suggested that transmission peaks at 8.72°C, based on counting the number of confirmed cases and temperatures worldwide. Sajadi et al. [18] discovered that the transmission of outbreaks was the highest at temperatures between 4°C and 11°C and relative humidity levels between 47% and 79%. Elevated temperatures inhibit the spread of SARS-CoV-2 [19, 20]. For example, Shaman et al. [21] discovered that an increase in autumn mortality due to SARS-CoV-2 in the UK in 2020 was associated with seasonality. However, Briz-Redon and Serrano-Aroca [22] reported controversial findings regarding the effect of temperature, possibly reflecting spatial heterogeneity in the relationship between temperature and SARS-CoV-2 transmission. Additionally, the effects of humidity [10, 19, 23], wind speed [20, 24], and atmospheric pressure [19, 25] have been investigated. UV radiation was shown to inhibit the spread of an epidemic. Most of these studies were conducted on a global scale. However, the Chinese region has unique climatic and regional features, and epidemic prevention and control should be established based on regional characteristics. Therefore, a regional analysis based on previous studies is needed.

According to the principle of epidemiological transmission, physical isolation is the most direct and effective method for preventing and controlling SARS-CoV-2 [26–28]. Most countries have implemented a series of non-drug interventions [29] to effectively control the spread of SARS-CoV-2 [30, 31]. Carleton et al. [25] reported that the effect of climate is less significant than that of physical isolation. However, policies and regulations impose direct effects only in the short term. By contrast, weather factors can have indirect long-term effects.

The SARS-CoV-2 spread worldwide, which significantly affected the economies and lives of residents in various countries. Different policy strategies have been developed in multiple regions in response to this crisis; however, these strategies are limited. For this study, China is selected as the study area and the provincial administrative region as the smallest organizational unit. The specific humidity (H), 2-m temperature (T), total precipitation (TP), ultraviolet (UV) radiation, wind speed (WS), and surface pressure (SP) are selected as weather factors to analyze the spatio-temporal distribution characteristics. The effects of weather factors on SARS-CoV-2 should be investigated to facilitate efforts toward long-term epidemic prevention.

## Material and methods

### Study area

**Fig 1:**
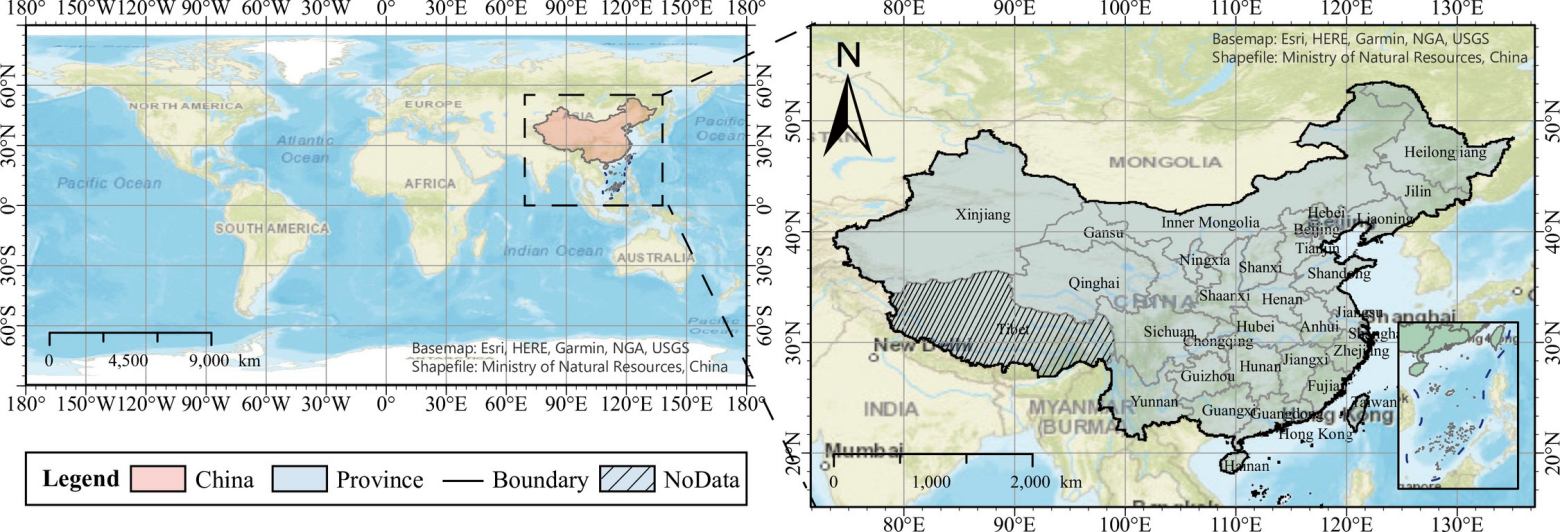
Study area. No data regions: Tibet, Hong Kong, Macau, and Taiwan.

China has a complex and diverse climate spanning tropical, subtropical, warm temperate, middle temperate, and cold temperate zones from south to north. The eastern region features a wide range of monsoon climates; the continental monsoon prevails in the winter, which is cold and dry; and the marine monsoon prevails in the summer, which is hot, humid, and rainy. The high altitude and large area of the Tibetan Plateau results in a distinct alpine climate. The northwestern region is located inland and is not affected by the marine monsoon; therefore, it has a westerly inland arid climate. Different climatic zones exhibit different weather characteristics, and the rich weather characteristics in China are beneficial for investigating the effects of weather factors on the SARS-CoV-2 outbreak (as shown in Fig 1).

The SARS-CoV-2 epidemic spread worldwide, which significantly affected the economies and lives of people in various countries. Various policy strategies have been developed in regions in response to address this crisis. China’s active and effective epidemic prevention efforts as well as timely macro-policy adjustments have inhibited the spread of the outbreak initially. However, China’s diverse climate, large and dense population, and complex living environment rendered the prevention and control of the outbreak more difficult, and the current problems have not yet been solved.

### Data sources

#### Time interval

**Fig 2:**
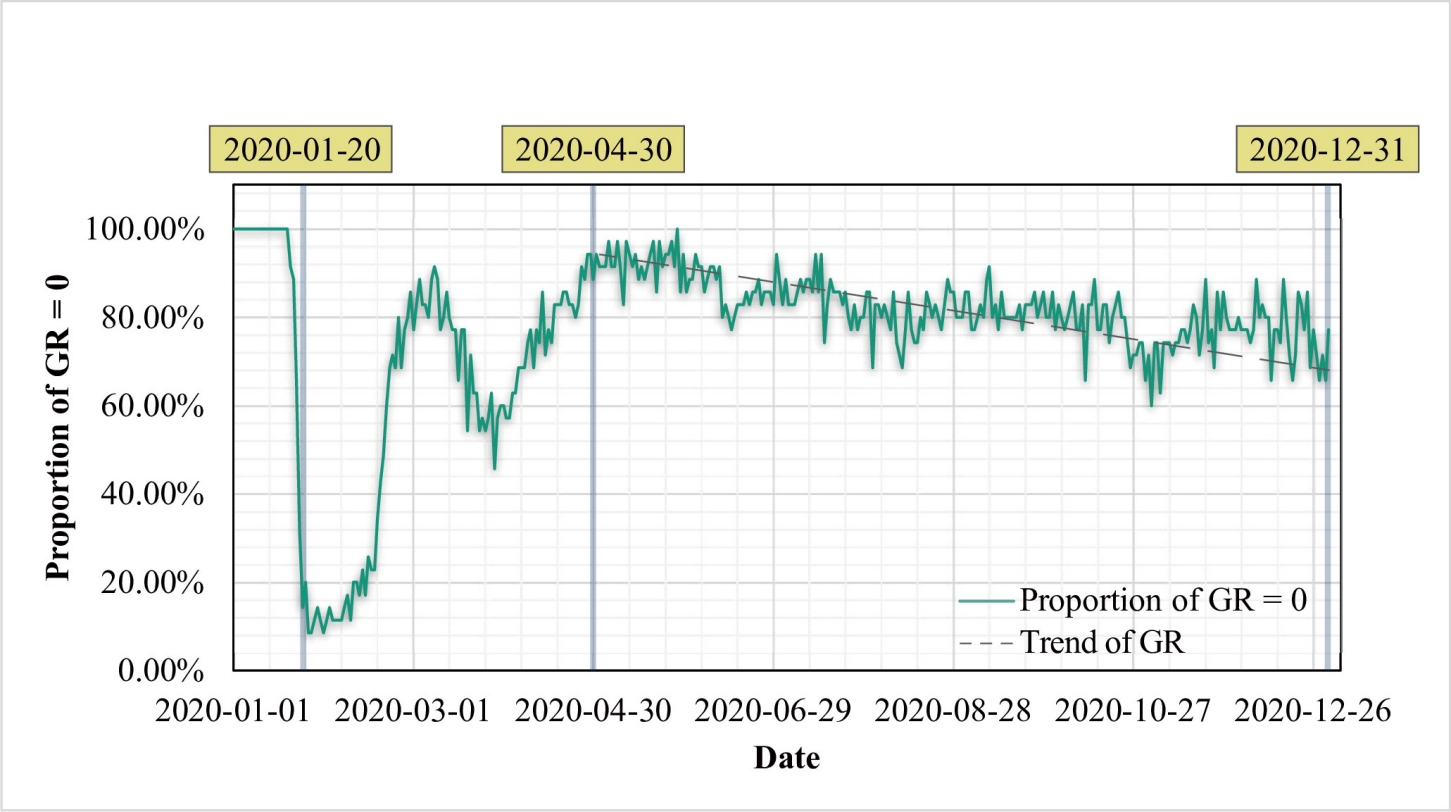
Percentage of provinces with zero growth rate in SARS-CoV-2. Notes: Statistics conducted by provincial administrative regions.

The SARS-CoV-2 growth rate (GR) of each provincial administrative region for year 2020 is shown in S1 Fig. The epidemic stabilized gradually around April 30, i.e., its rate of change increased or decreased less. The proportion of SARS-CoV-2’s zero GR in 2020 to the statistical data of all provinces in China, which show cyclical changes, is presented in Fig 2. Data from the first cycle for analysis, i.e., from January 20 to April 30, 2020 (a total of 101 days), were selected. This cycle was a period of high epidemic incidence and was affected less by policies and regulations. Furthermore, human control was less, and the analysis results of the epidemic GR and climatic factors in this cycle showed relatively objective and accurate results.

#### Epidemic data

Statistics pertaining to SARS-CoV-2 were obtained from the official website of *the National Health Commission of the People’s Republic of China*, including the cumulative number of confirmed, newly confirmed, existing confirmed, cured, newly cured, death, and recent death cases. The primary indicator used in this study was the cumulative number of confirmed cases (Confirmed).

#### Population data

The total population was obtained from *the China Statistical Yearbook (2020)*, which was based on the population at the end of 2019. The 2019 population density data obtained from LandScan were used [32].

#### Weather data

Weather data were obtained from the ERA5 reanalysis product of the European Center for Medium-Range Weather Forecasts (ECMWF) with a spatial resolution of 0.25° and an hourly temporal resolution. A total of six weather factors were selected (as shown in Table 1), namely 1,000 hPa hourly downward UV radiation at the surface (J/m^2^·h), 1,000 hPa H (%), T (°C), TP (mm), 10-m WS (m·s^-1^), and SP (kPa). The T, TP, SP, and WS data were obtained from ERA5-Land hourly data (from 1950 to the present), and the UV radiation and H data were obtained from ERA5 hourly data on pressure levels (from 1959 to the present). Among them, the UV radiation referred to solar radiation in the wavelength band between 100 and 400 nm, which is typically classified into three categories, i.e., UV-A (320–400 nm), UV-B (280–320 nm), and UV-C (100–280 nm). UV-B was used in this study. The WS in the data was calculated from the 10-m u-component and 10-m v-component of the wind, and the combined method for calculating *u* and *v* components is shown in Eq (1).


$$WS=\sqrt{{\left(u\right)}^{2}+{\left(v\right)}^{2}}$$ (1)

**Table 1:** Data information.

Name	Original Units	Units	Short Name	Abbreviation	Param ID	Number of data (scenes)	Format	Dataset
2-meter temperature	K	°C	2t	T	167	2,424	NetCDF	ERA5-Land hourly data from 1950 to present
Total precipitation	m	mm	tp	TP	228	2,424	NetCDF	
Surface pressure	Pa	kPa	sp	SP	134	2,424	NetCDF	
10-meter u-component of wind	m·s^-1^	m·s^-1^	u10	WS	165	2,424	NetCDF	
10-meter v-component of wind	m·s^-1^	m·s^-1^	v10		166	2,424	NetCDF	
Downward UV radiation at the surface	J·m^-2^	kJ·m^-2^·hour^-1^	uvb	UV	57	2,424	NetCDF	ERA5 hourly data on pressure levels from 1959 to present
Specific humidity	kg·kg^-1^	%	q	H	133	2,424	NetCDF	

Note: The selected 6 weather factors are included.

## Methods

### Data preprocessing

Data preprocessing is essential for investigating the correlation between the SARS-CoV-2 epidemic and weather factors, which primary determines whether credible results can be obtained.

#### (1) Data format conversion

The ECMWF data format was NetCDF (127 GB in total). Python’s netCDF4 package was used to convert it into hourly GeoTIFF format (16,968 views in total), convert the geographic coordinates into projection coordinates, establish a 10 km buffer at the boundary of China’s administrative regions, and then extract the weather data via the mask.

#### (2) Conversion of hourly data into daily data

For the TP, the daily data were summed by the hour; for other factors, the daily data were averaged by the hour; and the data processed resulted 606 views. Meanwhile, the units of the data were converted: the original units of SP was Pa, which was converted to kPa after dividing by 1,000; the original units of H was kg·kg^-1^, which was converted to percentage after multiplying by 100; the original units of UV was J·m^-2^, which was converted to J·m^-2^·h^-1^ after obtaining the average by the hour, and was converted to kJ·m^-2^·hour^-1^ after dividing by 1,000.

#### (3) Integrating population density and weather factors

SARS-CoV-2 is closely related to population distribution and can be used to obtain weather factor data under the effect of human activities. The resolution of the weather data was 0.25° × 0.25°, which was much lower than that of the population data (30’’ × 30’’). The weather data were resampled to the population resolution to avoid missing values at the boundary caused by multiplying the weather and population data. The LandScan population distribution was used as the weight, and the weather data were calculated to obtain the weighted weather data. The weather data featured a lower resolution, which eliminated the effect of mixed pixels and included the characteristics of human activities, as shown in Eq (2).

$$W_{it}=\sum_{g\in i}\omega_{g}W_{gt}$$ (2)

where ω is the population distribution weight, *W* the weather variable, *i* the provincial administrative region, *g* the grid in administrative region *i*, *t* the specified time, and *gt* grid *g* at time *t*. Finally, ArcMap’s zonal statistics were used to calculate the weighted weather data for each provincial administrative region. A comparison of the effects before and after data weighting is shown in S2 Fig. The SP, TP, UV, and WS were highly consistent before and after processing, whereas T and H were relatively sensitive to the population density, particularly H, indicating that human activities imposed a greater effect on these variables.

#### (4) Elimination of cross-sectional differences (decentralization)

**Fig 3:**
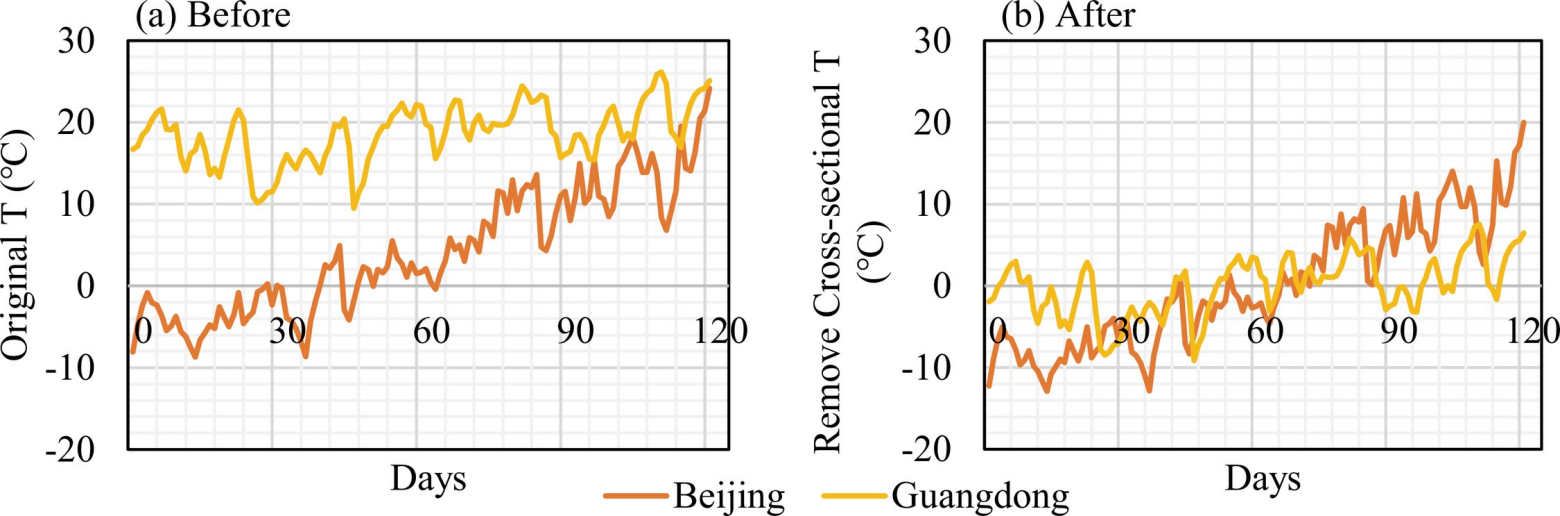
Data comparison before and after processing. Notes: Original vs. removed cross-sectional data. Beijing and Guangdong are used as examples.

The cross-sectional data refer to data obtained from different study areas at a specific time and correspond to a one-dimensional dataset composed of other spatial objects simultaneously. They allow a phenomenon at a particular time to be investigated and highlight the differences between spatial objects. The salient feature of cross-sectional data is their high dispersion. Additionally. cross-sectional data highlight individual differences and typically show irregular instead of actual random variations, i.e., unobservable heterogeneity. When analyzing cross-sectional data, to avoid cross-sectional differences between different regions from affecting the calculation results, the data of provincial administrative regions were decentered to eliminate fixed effects. The comparative effects of the data before and after the removal of the cross-sectional differences are shown in Fig 3.

### Change rate analysis

#### (1) GR of SARS-CoV-2

Carleton et al. [25] estimated a longitudinal (i.e., panel) regression model for daily confirmed cases in 173 countries by defining the GR of the SARS-CoV-2 epidemic as the daily GR of the administrative region. Regional differences did not affect the difference between the logarithm of confirmed cases on days *t* and *t*-1 in the administrative region, as shown in Eq (3), where *λ* is the growth rate, *C* the number of confirmed cases, *t* the specified time, and *i* the provincial administrative region.


$$\lambda_{t}^{i}=\text{ln}\left(C_{t}^{i}\right)-\text{ln}\left(C_{t-1}^{i}\right)$$ (3)

#### (2) Change rate of weather factors

The Theil-Sen slope (*TS*_*Slope*_) estimation is a non-parametric estimator for estimating the change rate of time-series data and was used in this study to characterize the long-term trend of weather factors. This estimation can be used for censored regression models and is insensitive to outliers. It can be more accurate than non-robust simple linear regression for skewed and heteroscedastic data and is comparable to non-robust least-squares methods, even for normally distributed data. In particular, for data with chaotic properties, this expression offers a clear advantage. *TS*_*Slope*_ is calculated as follows:

$$TS_{Slope}=median\left(\frac{x_{j}-x_{i}}{t_{j}-t_{i}}\right)$$ (4)

where the *median* is the median function, *x*_*i*_ and *x*_*j*_ are the series data, *t*_*i*_ and *t*_*j*_ are the times corresponding to the series data, *n* is the series length, and *j* is the serial number (1≤*i*≤*j*≤*n*). *TS*_*Slope*_ > 0 indicates an upward trend and the opposite indicates a downward trend. The higher the value of *|TS*_*Slope*_*|*, the more apparent is the trend.

### Contribution rate analysis

The Pearson correlation coefficient is typically used in correlation analyses to analyze linear correlations. Since a delay occurs between the initial SARS-CoV-2 exposure and infection and the effect of weather on the confirmed cases was not instantaneous, a lag occurred. The lag model was used to calculate the weather factors and GRs of the confirmed cases, then the cumulative effect model was used to calculate the contribution rate.

A lagged variable affects the explanatory variable in the past. The lagged variables include the lagged explanatory and explained variables. The lagged variables were substituted into the regression model, i.e., the lagged variable model. The distribution lag model was selected to quantify the degree of influence of weather factors on SARS-CoV-2, and the general expression is shown in Eq (5).

$$Y_{t}=\alpha +\beta_{0}X_{t}+\beta_{1}X_{t-1}+\beta_{2}X_{t-2}+\cdots +\beta_{s}X_{t-s}+u_{t}$$ (5)

where *s* is the lag length, *t* is the time, and each coefficient reflects the explanatory variable’s different degrees of influence of each lag value on the explained variable the multiplier effect. *β*_0_ is the short-term or immediate multiplier and represents the average size of the impact of a one-unit change in *X* on *Y* in the current period. *β*_*i*_ is the delayed or dynamic multiplier, which represents the average size of the effect of a one-unit change in *X* on *Y* in past periods. $\sum_{i}^{s}\beta_{i}$ is the long-term or total distribution multiplier, which represents the size of the full effect on *Y* due to the lag effect when *X* changes by one unit.

In the estimation process, multiple collinearities might be exhibited between explanatory variables, which can result in meaningless parameter estimation and failure to reveal *X*’s effect of each lag on the dependent variable. Therefore, polynomial distributed lag models, including the Almon distributed lag model [33, 34], were used to reduce the number of parameters to be estimated and smooth the lag coefficients. The six weather variables selected in this study were modeled with distribution lags, as shown in Eqs. (6), where *λ* is the SARS-CoV-2’s GR; *i* is the provincial administrative region; *t* is the specified time; *l* is the lag order (number of days); and UV, T, H, TP, WS, and SP are the weather variables.


$$\begin{array}{c} \lambda_{t}^{i}=\sum_{l=0}^{l=\text{L}}\alpha_{l}^{UV}UV_{i,t-l}+\sum_{l=0}^{l=\text{L}}\alpha_{l}^{T}T_{i,t-l}+\sum_{l=0}^{l=\text{L}}\alpha_{l}^{H}H_{i,t-l}+\sum_{l=0}^{l=\text{L}}\alpha_{l}^{TP}TP_{i,t-l}\\ +\sum_{l=0}^{l=\text{L}}\alpha_{l}^{WS}WS_{i,t-l}+\sum_{l=0}^{l=\text{L}}\alpha_{l}^{SP}SP_{i,t-l}+\theta^{\prime }Z_{it}+\epsilon_{it} \end{array}$$ (6)

### Spatial analysis

Using spatial autocorrelation methods, spatial analysis was performed to investigate whether the SARS-CoV-2 epidemic and weather factors in each provincial administrative region exhibited spatial clustering characteristics. Moran’s *I* is a typical spatial autocorrelation analysis method with strong applicability that can test whether a statistically significant spatial distribution exists and reveal the spatial process for generating this distribution [34], which can be classified into global and local. Global spatial autocorrelation assumes that the space is homogeneous, where only one trend fills the entire region [35]. It can only detect whether the research object has aggregated in the research region and cannot accurately determine the aggregation range. Therefore, local spatial autocorrelation is required to determine the specific aggregation region and range.

The local Moran’s *I* is a decomposition into individual region cells, and for a particular spatial cell *i*, its local Moran’s *I* is defined as shown in Eq (7).

$$I_{\text{i}}(d)=Z_{\text{i}}\sum_{\text{j}=1}^{\text{n}}W_{\text{ij}}Z_{\text{j}}$$ (7)

where *Z*_*i*_ and *Z*_*j*_ are the degrees of deviation of the observations from the mean, *W*_*ij*_ is a spatial weight matrix in row-normalized form, and *I*_*i*_ is the product of *Z*_*i*_ and the weighted average of the observation cells around spatial cell *i*.

## Results

### Spatial and temporal distribution characteristics

#### SARS-CoV-2 spatial distribution characteristics

**Fig 4:**
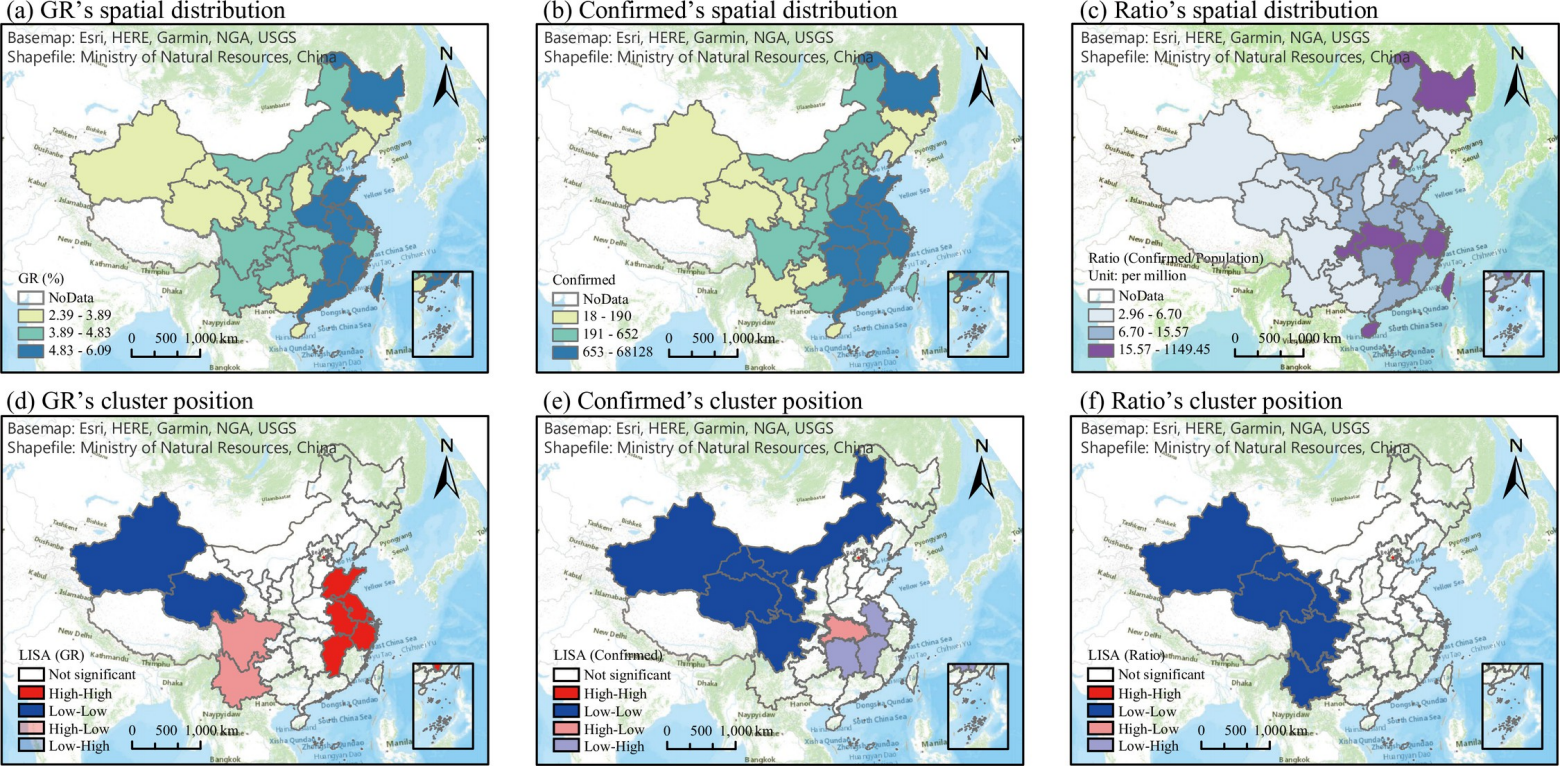
Spatial distribution and cluster position of SARS-CoV-2. Notes: (a) & (d) show spatial distribution of GR and cluster position; (b) & (e) show confirmed cases; and (c) & (f) show the ratio. GR is the growth rate of SARS-CoV-2 in China, “confirmed” is the number of confirmed cases, and the ratio is the percentage of confirmed cases in the population.

In the spatial distribution characterization of the GR, Xinjiang and Qinghai were in the low-low (LL) cluster; Yunnan and Sichuan were in the high-low (HL) cluster; and Shandong, Anhui, Jiangsu, Jiangxi, and Zhejiang were in the high-high (HH) cluster. All of the regions were significant statistically (*P-value* < 0.05), and no low-high cluster regions were discovered. Xinjiang and Qinghai were surrounded by provinces with higher GRs. Yunnan and Sichuan were surrounded by provinces with lower GRs, whereas the provinces in the HH aggregation region had higher GRs than the surrounding provinces, as shown in Fig 4.

Based on the confirmed spatial distribution, Hubei showed HL clustering; Anhui, Hunan, and Jiangxi showed low-high (LH) clustering; and Gansu, Inner Mongolia, Qinghai, Sichuan, and Xinjiang showed LL clustering, all of which were significant significantly (*P-value* < 0.05), and no areas of HH clustering were discovered. The number of confirmed cases in Hubei Province was much higher than the average number among all regions in China. The number of surrounding sections was lower than the number of confirmed cases in Hubei, indicating significant HL clustering (*P-value* < 0.05). The northern region of China, with a relatively small population and few confirmed infection cases, showed LL clustering. Three provinces (Anhui, Hunan, and Jiangxi) are adjacent to Hubei Province and are connected. The number of confirmed cases in these three immediate provinces was higher than that in Hubei Province, indicating significant LH clustering.

The Hu Line segregates the level of population from the social and economic pattern in China, from Heihe City, Heilongjiang Province, to Tengchong City, Yunnan Province, where approximately 44% of the country’s territory southeast of the line is inhabited by approximately 94% of the population [36]. In general, it delineates the urbanization levels, with most of the southeastern provinces and cities along this line exhibiting urbanization levels higher than the national average. Generally, both the GR and confirmed spatial distribution characteristics were consistent with the Hu Line, which indicates that the SARS-CoV-2 epidemic was more severe in cities with higher population concentrations and urbanization levels.

#### SARS-CoV-2 temporal variation characteristics

**Fig 5:**
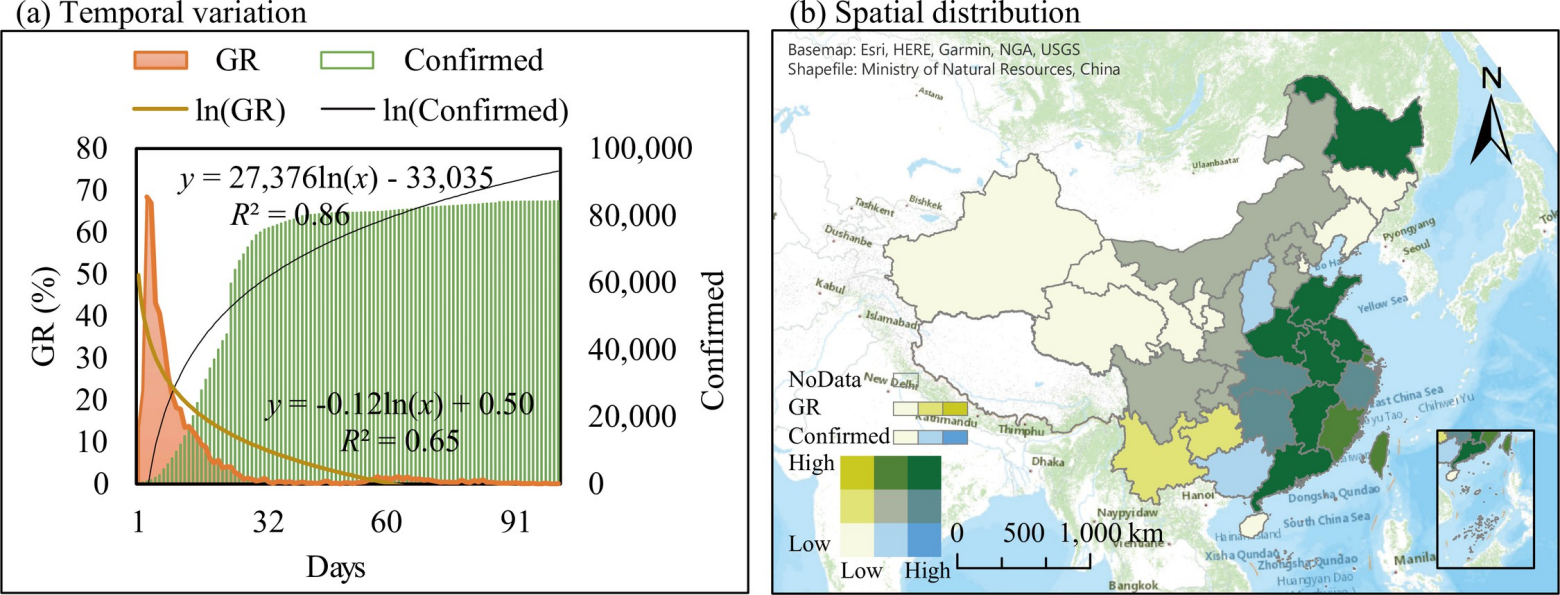
Distribution characteristics of number of confirmed SARS CoV-2 cases and its growth rate in China. (a) Temporal variation; (b) spatial distribution.

The average GR of all provinces (GR = 5.15%) showed an overall logarithmic distribution with a significant downward trend (*P-value* < 0.05), with 84,385 confirmed cases as of April 30 and an average of 833 new cases per day (Fig 5). The highest GR was recorded on January 23, 2020 (GR = 68.47%), with 870 confirmed cases and 290 new confirmed cases, and the average GR for eight days from January 23 to January 31 was 37.43%. From January 27 to February 18, the number of newly confirmed cases exceeded 1,000 per day, with an average GR of 14.32%.

The highest GR values were recorded from January 20 to January 27 (eight days), as shown in S3 Fig. For most provinces, the maximum GR was recorded within two days of the first confirmed case, i.e., the first and second days of the outbreak; subsequently, it decreased for the three provinces (Hubei, Jiangxi, and Xinjiang). These effects appeared only after the outbreak. For Hubei and Jiangxi, the severity of the epidemic was indirectly demonstrated by the proximity of the two provinces and the intense population circulation. Xinjiang indirectly demonstrated the difficulty in controlling the epidemic owing to the lack of economic development and convenient transportation.

Hubei Province recorded the highest number of confirmed cases, with 68,128 confirmed cases as of April 30, constituting 80.73% of the total, whereas confirmed cases in the other 32 provinces (excluding Tibet) constituted only 19.27%. This was followed by Guangdong, Henan, Zhejiang, and Hunan with 1,588, 1,276, 1,268, 1,037, and 1,019 confirmed cases, respectively, as of April 30, with a total of 6,188 confirmed cases, constituting 7.33%. The overall number of confirmed cases was 58.92 per million of the population, and the average for each province was 51.47 per million people. The highest ratio was observed in Hubei (1,149.45 per million). Although the number of confirmed cases in Guangdong, Henan, and Hunan provinces was relatively high, the proportion of confirmed cases in the population was relatively low. By contrast, provinces which indicated value above the average value included Beijing (27.53 per million), Shanghai (26.85 per million), Heilongjiang (25.17 per million), Zhejiang (21.68 per million), and Jiangxi (21.68 per million), Jiangxi (20.08 per million), Chongqing (18.53 per million), and Hainan (17.78 per million). Notably, the total provincial population, GR, and confirmed cases were not sufficient to allow a unilateral assessment regarding the severity of the regional epidemic.

#### Spatial and temporal variation characteristics of climatic factors

The long-term trends of the six weather factors were calculated based on the Theil-Sen trend. Based on the results, only the SP indicated a downward trend, whereas the remaining five factors showed an upward trend, as shown in Table 2. The statistical indicators for each factor are shown in S1 Table. Among them, T and UV showed a significant upward trend (*P-value* < 0.05), whereas the WS and TP fluctuated significantly, although the trend was not significant (*P-value* > 0.05). The values of T and UV were 0.15°C/day and 0.56 kJ·m^-2^·h^-1^/day, respectively. The long-term change trend indicates that the GR and SP decreased simultaneously, whereas the GR and the other five factors showed the opposite trend; the statistics of each province are shown in S4 Fig.

**Table 2:** Theil-Sen change trend of various weather factors.

	H	T	TP	UV	WS	SP
**CR (unit/day)**	2.59E-03	0.15	0.01	0.56	4.30E-03	-0.01
**Significant**	-	*	-	*	-	-

Note: * represents *P-value* < 0.05. The Theil-Sen change rate describes a long time series that differs from the growth rate (GR). The GR represents the change rate of two time points, which can be regarded as the short-term change rate.

**Fig 6:**
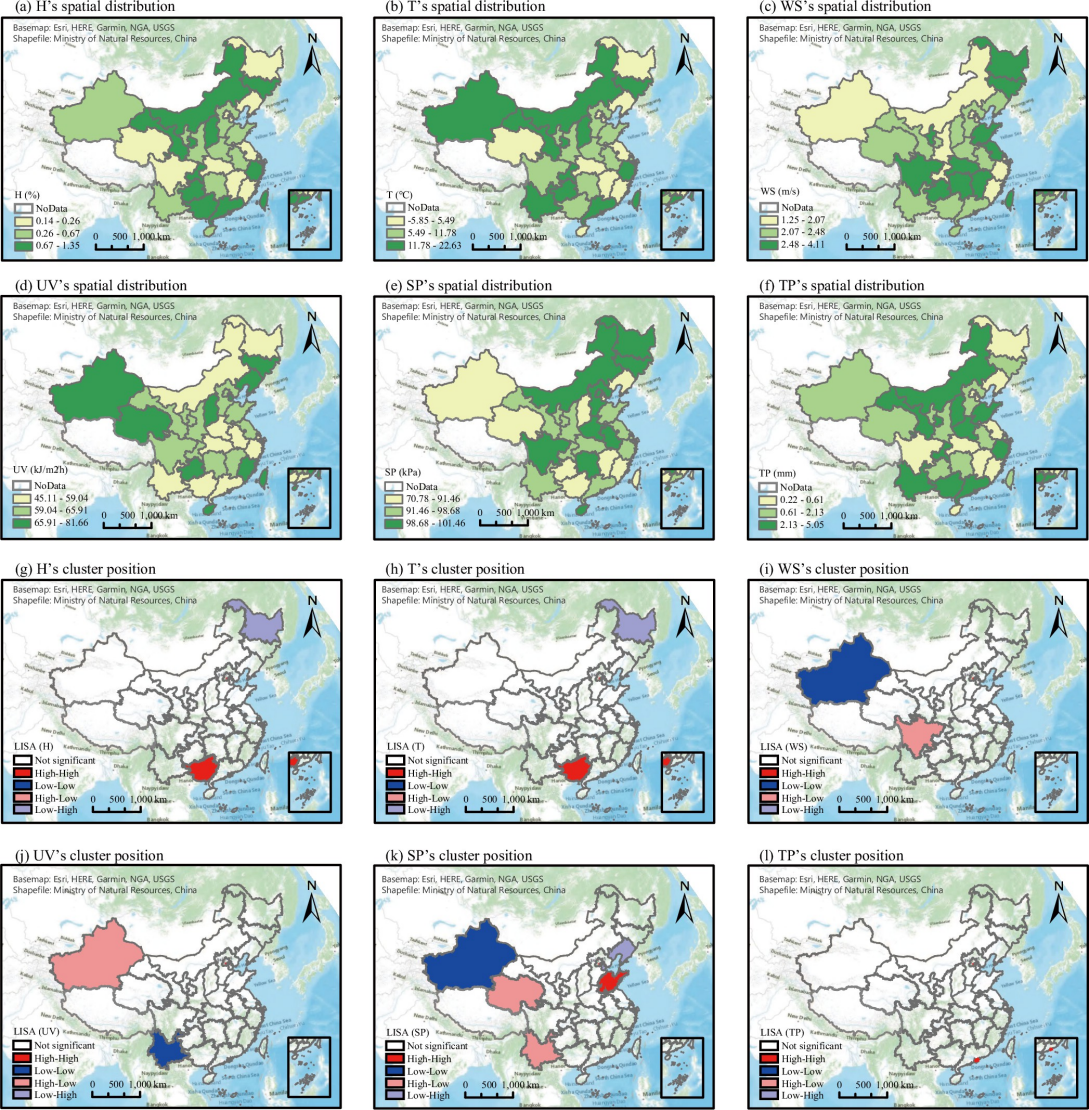
Spatial distribution characteristics of weather factors. Note: growth rate of SARS-CoV-2 (GR), specific humidity (H), 2-m temperature (T), wind speed (WS), ultraviolet (UV) radiation, surface pressure (SP), and total precipitation (TP).

The spatial distribution and cluster characteristics of each factor are shown in Fig 6. H and T showed consistent cluster characteristics, with HH and LH clusters in Guangdong and Heilongjiang, respectively. UV and WS showed opposite cluster characteristics, with an LL cluster in Yunnan and an HL cluster in Xinjiang for UV, and a LL cluster in Xinjiang and an HL cluster in Sichuan for WS. In terms of the SP, Xinjiang was an LL cluster, Shandong was an HH cluster, Liaoning and Tianjin were LH clusters, and Yunnan and Qinghai were HL clusters.

### Relationship between climatic factors and SARS-CoV-2

#### Correlation of SARS-CoV-2 with weather factors

Among the correlations between UV and the other five factors, all were significant (*P-value* < 0.05), except for WS, whereas UV indicated the lowest correlation with WS (R_WS_ = 0.13). UV was significantly negatively correlated with GR, TP, and SP, whereas the other parameters were positively correlated with them. UV showed the highest correlation with T (R_T_ = 0.87), followed by SP (R_SP_ = -0.55), and GR (R_GR_ = -0.53), as shown in S5 and S6 Figs and S2 Table.

Among the correlations between GR and the six weather factors, all were significant (*P-value* < 0.05) except for TP, which was positively correlated with TP and SP (R_TP_ = 0.14, R_SP_ = 0.35), whereas the remaining four factors were significantly negatively correlated with TP and SP, where GR indicated the highest correlation with UV and T (R_UV_ = -0.53, R_T_ = -0.51), followed by P and WS (R_P_ = -0.33, R_WS_ = - 0.23).

In addition, T indicated a strong positive correlation with P and a strong negative correlation with SP, both of which were significant (*P-value* < 0.05). T showed a weak positive correlation with WS, which was significant (*P-value* < 0.05). No correlation was indicated with TP. P indicated a significant negative correlation with SP (*P-value* < 0.05), whereas the remaining factors were positively correlated with SP. TP indicated a strong positive correlation with WS, whereas its correlations with the other factors were weak. Except for P and UV, SP showed weak negative correlations with other factors.

**Fig 7:**
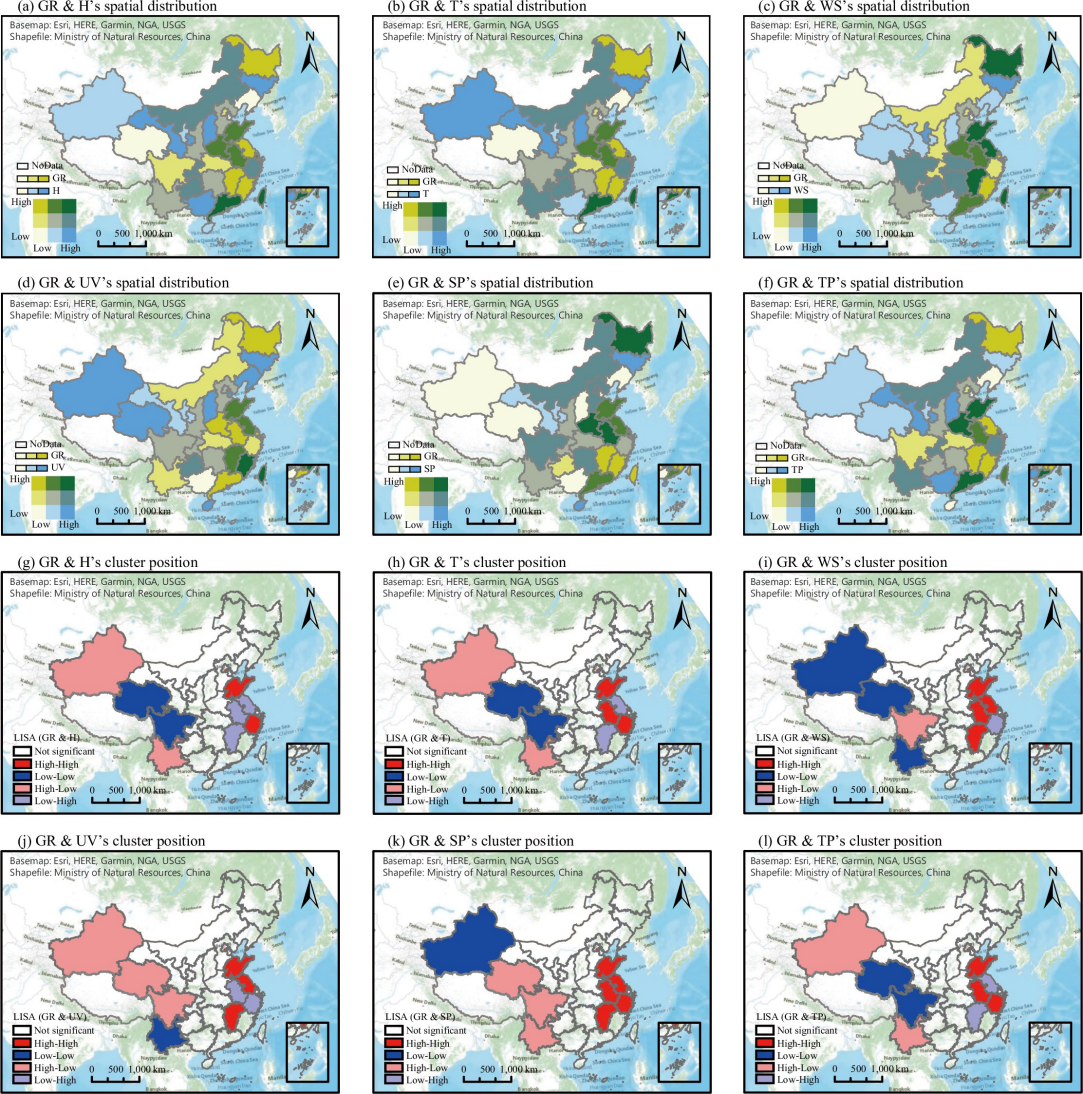
Spatial distribution correlation between growth rate (GR) of SARS-CoV-2 and each weather factor. Notes: Overlay analysis and spatial cluster analysis of GR and weather factors were included.

The spatial distribution and cluster characteristics of the GR and each weather factor are shown in Fig 7. In terms of the GR and weather sensitivity, Guangdong and Guangxi indicated the characteristics of LL and LH clusters, respectively, indicating that the regions around these provinces showed high climate sensitivity. The cluster distribution characteristics of the GR and weather factors can be categorized into two regions, eastern and western China, with the eastern region showing HH and LH cluster characteristics, and the western region showing LL and HL clusters. Neither northern nor central China showed spatial cluster characteristics, which indicates that weather factor responses to the epidemic were evident in western and eastern China.

#### Weather sensitivity analysis

**Fig 8:**
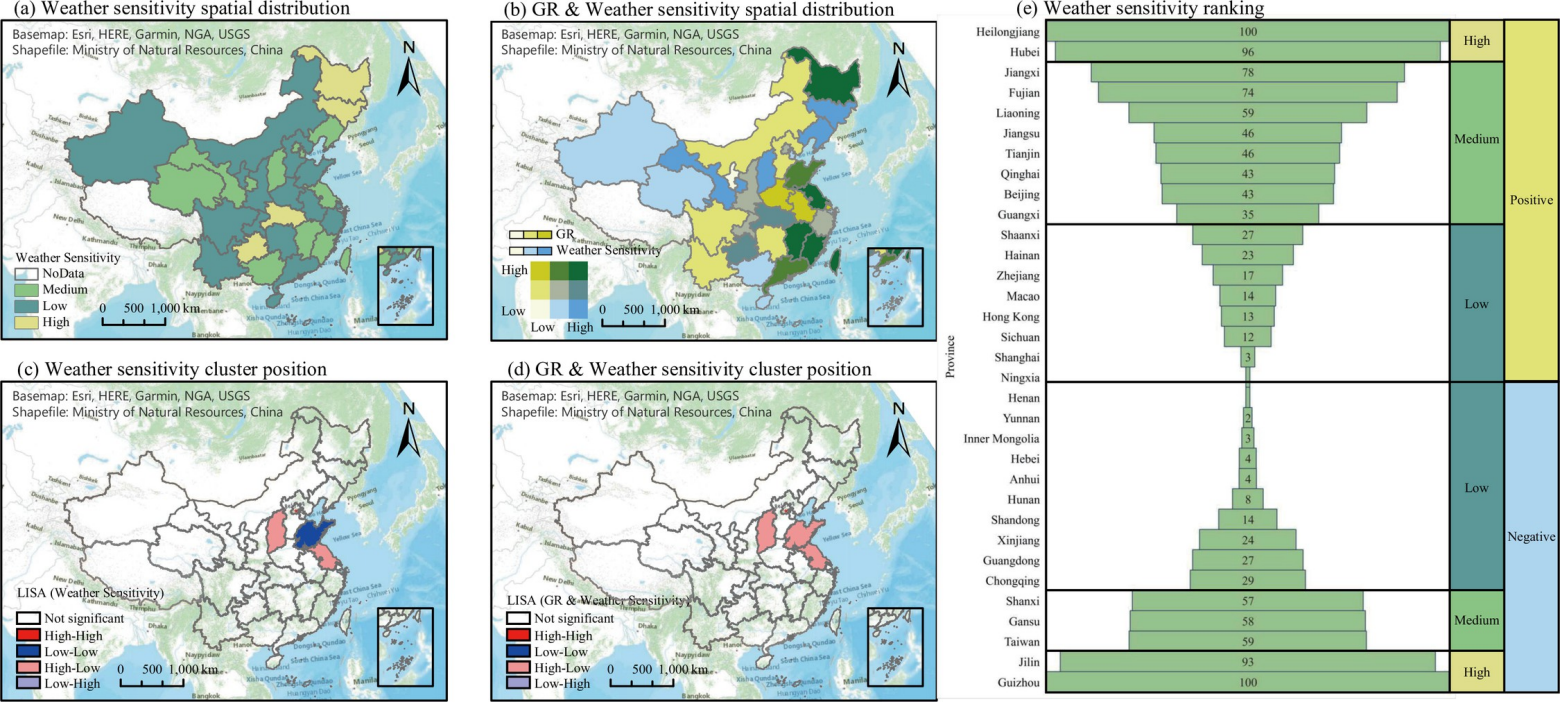
Distribution and ranking of weather sensitivity. Note: To determine weather sensitivity, principal component analysis was performed to calculate the comprehensive scores of six weather factors (UV, H, TP, WS, SP, and T), which were mapped to values of 1 to 100; subsequently, K-means clustering was used to classify them into three types (high, medium, and low). The comprehensive score (before mapping) included two sets, higher or lower than zero, which represent positive and negative directions, respectively. A value closer to zero significances less significant effect.

Six weather factors were selected, and the individual analyses presented certain limitations. To investigate the overall effect of weather factors on the SARS-CoV-2 epidemic, the six weather factors were represented in the form of a composite score for each province using principal component analysis, which was then classified into high, medium, and low levels using the K-means clustering algorithm. These levels represent the weather sensitivity, i.e., the level by which each region was affected by the weather. The spatial distribution of weather sensitivity is shown in Fig 8A, and the composite score of each province is shown in Fig 8B. Heilongjiang, Jilin, Hubei, and Guizhou were high-sensitivity provinces; 11 provinces were of medium sensitivity; and the remaining 18 provinces were low-sensitivity regions. LL clusters were indicated in Shandong, whereas HL clusters were indicated in Jiangsu and Shanxi. No cluster characteristics were discovered in other provinces, and the overall cluster characteristics were not significant (*P-value* > 0.05).

Among the high-sensitivity regions, Heilongjiang and Hubei were classified as Group A, and Jilin and Guizhou were classified as Group B. The T in Groups A and B was low and high, respectively; the TP in Groups A and B was low and medium, respectively; the WS of all four regions were high; the SP of Heilongjiang and Jilin was high, whereas those of Hubei and Guizhou were medium and low, respectively, which is attributable to altitude. The remaining three regions, except Heilongjiang, did not indicate high GRs; in particular, Groups A and B indicated high and low levels, respectively. Although Heilongjiang is adjacent to Jilin (in northern China), and Hubei and Guizhou are in close proximity to each other (in southern China), their spatial and temporal characteristics were different. Additionally, the spatial distribution of the epidemic was not directly related to the south and north of China. Except for the eastern coastal region of China and the medium- and low-sensitivity areas, the GR and confirmed GR were at medium to low levels in most provinces, indicating a positive relationship between weather sensitivity and GR. The eastern coastal region is economically developed, with a dense and mobile population dominated by human activities, where weather is only a secondary factor. Therefore, physical isolation was the most effective measure for controlling the outbreak and spread of the epidemic in this region.

### Contribution rate of weather factors to SARS-CoV-2

The GR was lagged with each weather factor to obtain the impact values of the individual factors. In the 36^th^ lag analysis, WS and SP lagged by 11, UV, T, and H lagged by 8, 9, and 10, respectively, and TP lagged 17. In the multifactor analysis, the mean lag days of the six weather factors were used to obtain a total lag of 11 days, and the validation showed that the lag order of the GR was consistent with the simultaneous consideration of the six weather factors.

In the single-climate factor analysis, for every one unit increase in each factor, the GR decreased by 23.29±3.00% in 10 days (in H, *P-value* < 0.001), decreased by 0.56±0.08% in 9 days (in T, *P-value* < 0.001), increased by 0.67±0.31% in 17 days (in TP, *P-value* = 0.002 < 0.005), decreased by 0.17±0.03% in 8 days (in UV, *P-value* < 0.001), decreased by 12.01±0.02% in 11 days (in WS, *P-value* < 0.001), and increased by 4.81±1.02% in 11 days (in SP, *P-value* < 0.001).

In the multiclimate factor analysis (in 11 days), the GR decreased by 16.61±20.28% for each 1% increase in H; 1.58±1.11% for each 1°C increase in T; 0.90±0.50% for each 1 mm increase in the TP; 0.30±0.21% for each 1 kJ·m^-2^·h^-1^ increase in UV radiation; 16.47±2.93% for each 1 m·s^-1^ increase in the WS; and 15.02±1.63% increase in the GR for each 1 kPa increase in the SP. The model interpretability was 0.85 (*P-value* < 0.001) and the coefficients of each factor are shown in S3 Table.

**Fig 9:**
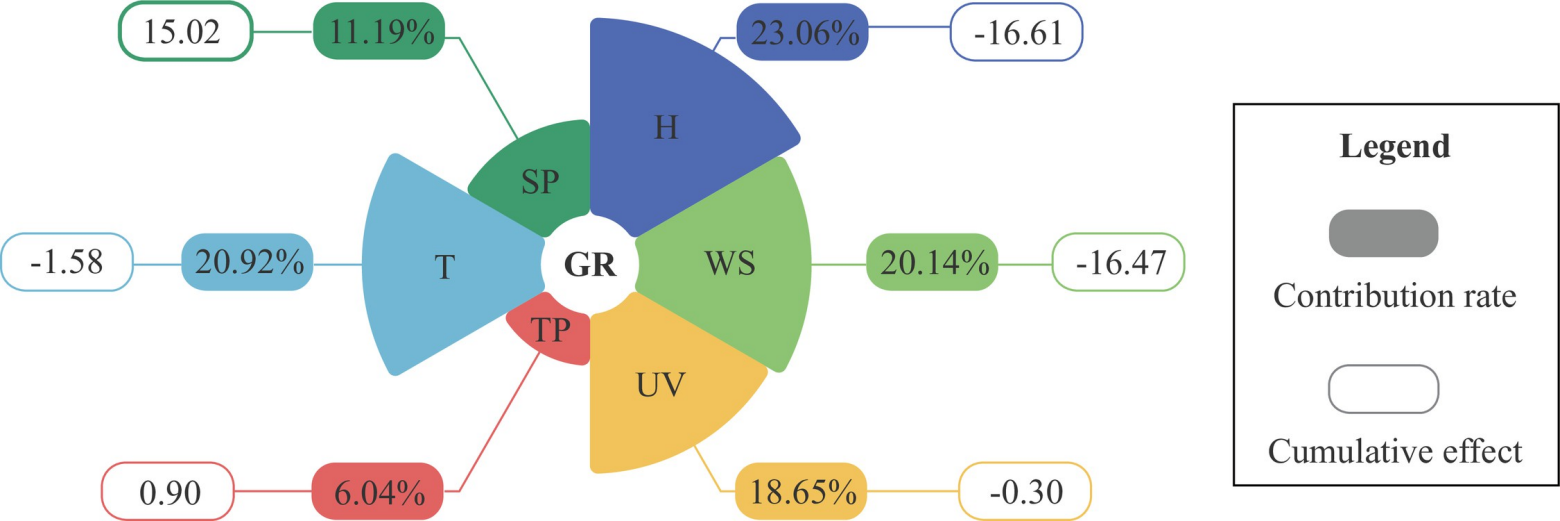
Effects of various weather factors on SARS-CoV-2. Notes: GR is the growth rate of SARS-CoV-2, H is specific humidity, T is 2-m temperature, WS is wind speed, UV is ultraviolet, SP is surface pressure, and TP is total precipitation.

Among the six weather factors, H exerted the greatest effect (23.06%), followed by T (20.92%) and UV (18.65%). The effect of TP was negligible at 6.04%, and the correlation between TP and GR was minimal, indicating that the effect of TP was insignificant; the effect of each factor is shown in Fig 9.

## Discussion

### Spatial and temporal differences

#### Regional differences caused by weather

The first case of SARS-CoV-2 was discovered in Wuhan, Hubei Province, on December 8, 2019, and the first case of SARS was discovered in Foshan, Guangdong Province, in November 2002, which were very similar in terms of the outbreak seasons. Previous studies [29] demonstrated that natural factors, particularly meteorological conditions such as temperature, precipitation, and radiation intensity, change the occurrence and spread of infectious diseases by affecting the pathogens, hosts, and transmission environments. Extreme weather events such as high temperatures, droughts, and floods pose direct risks to human health and indirectly affect the epidemic intensity and scope of infectious diseases [37].

The Chinese region includes six climatic zones: tropical, subtropical, warm temperate, middle temperate, cold temperate, and plateau climatic zones (as shown in S7 Fig). China has a distinctly monsoonal climate, and the seasonal distribution of precipitation varies widely, with the rainy season occurring from April to September in the south and July to August in the north, whereas the winters are relatively dry. Drought may affect the spread of viral infections by damaging the ecological environment. The adverse effect of precipitation on water quality and health during drought periods has been suggested to be higher than that of temperature [38]; additionally, drought can increase water pollution, causing increased pollution concentrations in river runoffs, deteriorating water quality, and affecting human health [39]. High temperatures and droughts can cause atmospheric pollution. Historically, many cases of drought-induced ecological damage has resulted in epidemic outbreaks [37]. In the analysis of the spatial distribution characteristics of the SARS-CoV-2 epidemic, the infection rate was higher in provinces located in warm temperate, subtropical, and tropical zones, which is consistent with the findings of many previous studies [40]. Not only for SARS-CoV-2, but also for most infectious diseases, the weather characteristics of temperate and tropical regions are more likely to be favored by the virus. The distribution characteristics coincided with those of the Hu Line. From an ecogeographic perspective, the Hu Line segregates the arid zone from the humid zone. The recently discovered 400 mm isoprecipitation line coincides with the Hu Line and revealed a high correlation between weather and population density [36].

#### Effect of urbanization on SARS-CoV-2

Population density has been shown to be an essential factor that determines the incidence of some infectious diseases, with a high incidence associated with high population density [41]. According to the fifth Chinese census in 2000, the eastern side of the Hu Line comprised approximately 94% of the population in China, whereas the western side comprised only approximately 6% of the population, which is consistent with the spatial distribution of epidemic severity.

The Hu Line segregates the level of population density from the social and economic patterns in China. Furthermore, it segregates the urbanization levels (as shown in S7 Fig). After more than half a century of development, the population in west China increased by only 2%, and the economic disparity widened. Most provinces and cities in the southeast indicated urbanization levels above the national average, whereas those of most regions in the northwest were below the national average. This is attributable to differences in the population density. However, it revealed the integrated pattern of natural, human, economic, and social factors in China, particularly the weather and landscape, which are important factors, thus resulting in a significant difference in the natural ecology. The spatial distribution of the GR in the southeastern region of China shows a more intense pattern compared with that in the northwestern region, which is consistent with the area delineated by the Hu Line. This indicates the effect of the urbanization level on SARS-CoV-2.

Owing to accelerated urbanization, rapid population and economic development, as well as the uncontrolled exploitation of the natural environment, humans are constantly considering the ecological resources of other organisms. Intense human activities have disrupted the balance of ecosystems, increased environmental risks and the migration of virus–host habitats, as well as increased the probability of viral mutations and the risk of infectious disease outbreaks. Simultaneously, the cluster of different viruses hosted after habitat migration significantly increases the probability of virus mutations and the risk of new infectious disease outbreaks. Studies have suggested that low precipitation and food shortages may cause animals to congregate around scarce resources, which increases host-to-host contact for viral transmission [42].

### Effect proportion of weather factors on SARS-CoV-2

Environmental and climate changes can significantly affect ecosystems. Their effects on the occurrence, transmission, and changes in infectious diseases are among of the most detrimental to health [43]. Infectious diseases comprise three main elements: pathogens, hosts, and transmission routes. Therefore, changes in climate or weather conditions may affect infectious diseases depending on the environment in which they exist [44]. Climatic conditions limit the geographic and seasonal distribution of infectious diseases, whereas environmental and meteorological factors may affect the timing and intensity of disease outbreaks [45].

According to the principles of epidemiological transmission, physical isolation is the most direct and effective method for disease control. The implementation of the home exclusion policy in China resulted in an effective control of person-to-person transmission of SARS-CoV-2. Policy regulations imposes a direct but short-term effect in preventing and managing the SARS-CoV-2 epidemic, whereas climatic factors imposes a long-term indirect effect. Pathologically, UV-C exerts an inhibitory effect on SARS-CoV-2 and has been used in disinfection procedures in hospitals and laboratories [16]. UV radiation is solar radiation in the wavelength band between 100 and 400 nm and is typically classified into three categories: UV-A (320–400 nm), UV-B (280–320 nm), and UV-C (100–280 nm). UV-C is mainly completely absorbed. A considerable amount of UV-A directly reaches the ground and can tan the skin, produce vitamin D, and affect photosynthesis. UV-B is mainly absorbed; however, even if this radiation reaches the ground in small amounts, it can cause significant environmental and ecological effects, as well as affect human health [46]. Carleton et al. [25] showed that for every one unit increase in UV radiation worldwide, the GR decreased by 13.2% over 17 days (0.78% per day). However, the UV impact in China was higher than the worldwide level, with a decrease of 18.65% over 11 days (1.70% per day), i.e., 0.92% higher than the world average per day.

Temperature was the most direct characterization indicator for each weather factor, and in Hubei, where the outbreak was the most severe. For example, temperatures from January 20, 2020 to April 30, 2020 indicated that Hubei province (particularly Wuhan city) was in the late winter and early spring season, with an average daily temperature range of 1°C to 18°C. In February, the average temperature in Wuhan City was within the optimal range for coronavirus transmission [17, 18]. Low temperatures and significant diurnal temperature differences resulted in a decrease in human immunity and ease of viral infection, thus facilitating the outbreak of SARS-CoV-2.

When atmospheric temperature variation was not considered, the SP decreased exponentially as the terrain increased, i.e., the SP was lower in higher-altitude regions. The SP increased gradually as the geographical latitude increased. The annual variation pattern of continental atmospheric pressure showed a higher SP in the winter than in the summer [47]. If only the effect of SP is considered, then highland regions such as Yunnan, Guizhou, and Tibet will indicate higher altitudes and lower SPs. Regions such as Guangdong, Shandong, Jiangsu, and Wuhan in Hubei had lower altitudes and higher SPs. Meanwhile, Heilongjiang was located in the northernmost region of China and belonged to the region with the highest latitude and higher SPs. The study was performed in the winter and early spring. The SP in the winter showed an overall decreasing trend and was lower than that in summer. Thus, it can be concluded that fewer people had SARS-CoV-2 when the SP was low. More confirmed SARS-CoV-2 cases were indicated when the SP was high. Therefore, a lower SP decreases the GR, which is contrary to the findings of Correa-Araneda et al. [19].

Many studies have shown that WS inhibits epidemics and infectious diseases such as SARS-CoV-2 [10]. Increasing the WS promotes indoor air circulation; however, wind can serve as a carrier of infectious disease pathogens. Therefore, wind direction and WS affect the spread and distribution of certain contagious diseases. The results showed that WS was negatively correlated with the GR and that the wind speed imposed an inhibitory effect on the GR.

In summary, the effect of weather on SARS-CoV-2 was demonstrated using spatio–temporal analysis. In addition, the effects of six weather factors (H, WS, SP, T, UV, and TP) on SARS-CoV-2 was quantified using statistical analysis to contribute to the prevention and control of the SARS-CoV-2 epidemic.

## Conclusion

The Chinese region was selected as the study area owing to its intense outbreaks and improved prevention and control. ECMWF reanalysis data were used for weather factors, and mathematical, statistical, and spatial analysis methods were used to analyze the temporal and spatial effects of the weather factors on the SARS-CoV-2 epidemic. Using the environmental and weather characteristics of infectious disease outbreaks for reference, the effects of six weather factors on the epidemic were discussed to facilitate the future prevention, control, interruption, and prediction of the epidemic. The main findings of this study are as follows:

1. In terms of spatial and temporal characteristics, the average GR of all administrative regions in China showed a logarithmic distribution with a significant downward trend (*P-value* < 0.05). The average GR was 5.15%, and 84,385 confirmed cases were recorded as of April 31, with an average of 833 new confirmed cases per day. The ratio of confirmed cases to the population showed a characteristic consistent with the spatial distribution of the Hu Line.
2. In the correlation analysis, UV was significantly and negatively correlated with GR, TP, and SP, with UV having the highest correlation with T (R_T_ = 0.87), followed by SP (R_SP_ = -0.55) and GR (R_GR_ = -0.53). The GR was significantly and positively correlated with SP (R_SP_ = 0.35), insignificantly and positively correlated with TP (R_TP_ = 0.14), and significantly and negatively correlated with the other four factors. GR had the highest correlation with UV and T (R_UV_ = -0.53, R_T_ = -0.51). This indicates that UV, H, T, and WS imposed inhibitory effects on GR, whereas SP and TP facilitated the GR.
3. In terms of the effects of the weather factors, the most significant effect on GR was indicated by H (ratio = 23.06%), followed by T (ratio = 20.92%) and UV (ratio = 18.65%). For every 1% increase in H, every 1°C increase in T, and every 1 kJ·m^-2^·h^-1^ increase in UV, the GR decreased by 16.47±20.28%, 1.58±1.11%, and 0.30±0.21%, respectively. The UV effect in China was higher than the global level (0.92% higher per day). However, theoretically, climate or weather changes within normal ranges do not prevent the spread of SARS-CoV-2. The factors that directly affected the SARS-CoV-2 epidemic were high population aggregation and high-level urbanization, whereas weather imposed only an indirect effect.

## Supporting information

S1 Fig SARS-CoV-2 growth rate change trend in each province of China in 2020 (Unit: %). The green line is the growth rate of SARS-CoV-2 in each province, and the yellow line is April 30, 2020, which is used to select the time period.
(TIF)

S2 Fig Data comparison before and after processing. Notes: Original versus weighted data. Take Beijing and Guangdong for example. Abbreviations: growth rate of SARS-CoV-2 (GR), specific humidity (H), 2-meter temperature (T), wind speed (WS), ultraviolet (UV), surface pressure (SP), and total precipitation (TP).
(TIF)

S3 Fig Date distribution of the highest growth rate (GR) of SARS-CoV-2 in each province (Unit: %). (TIF)

S4 Fig Time series characteristics of various factors in each province. Notes: growth rate of SARS-CoV-2 (GR), specific humidity (H), 2-meter temperature (T), wind speed (WS), ultraviolet (UV), surface pressure (SP), and total precipitation (TP).
(TIF)

S5 Fig Correlation of various factors. Notes: growth rate of SARS-CoV-2 (GR), specific humidity (H), 2-meter temperature (T), wind speed (WS), ultraviolet (UV), surface pressure (SP), and total precipitation (TP).
(TIF)

S6 Fig Correlation between SARS-CoV-2 growth rate and each weather factor (time series). Notes: growth rate of SARS-CoV-2 (GR), specific humidity (H), 2-meter temperature (T), wind speed (WS), ultraviolet (UV), surface pressure (SP), and total precipitation (TP).
(ZIP)

S7 Fig Distribution of climate zones in China and Hu Line (Heihe-Tengchong Line). (TIF)

S1 Table Statistical information of each factor. Notes: growth rate of SARS-CoV-2 (GR), specific humidity (H), 2-meter temperature (T), wind speed (WS), ultraviolet (UV), surface pressure (SP), and total precipitation (TP).
(DOCX)

S2 Table Correlation of various factors. Notes: * represents *P-value* < 0.05, and · represents *P-value* < 0.01. Abbreviations: growth rate of SARS-CoV-2 (GR), specific humidity (H), 2-meter temperature (T), wind speed (WS), ultraviolet (UV), surface pressure (SP), and total precipitation (TP).
(DOCX)

S3 Table Coefficient of cumulative effect analysis. Notes: R-squared = 0.85, Adjusted R-squared = 0.81, and Prob(F-statistic) < 0.001. Abbreviations: growth rate of SARS-CoV-2 (GR), specific humidity (H), 2-meter temperature (T), wind speed (WS), ultraviolet (UV), surface pressure (SP), and total precipitation (TP).
(DOCX)
