# Supplementary figures and images for: Impact of climate change on SARS-CoV-2 epidemic in China
Source: PLoS One. 2023 Jul 27;18(7):e0285179. doi: 10.1371/journal.pone.0285179 (PMC10374073; doi:10.1371/journal.pone.0285179)

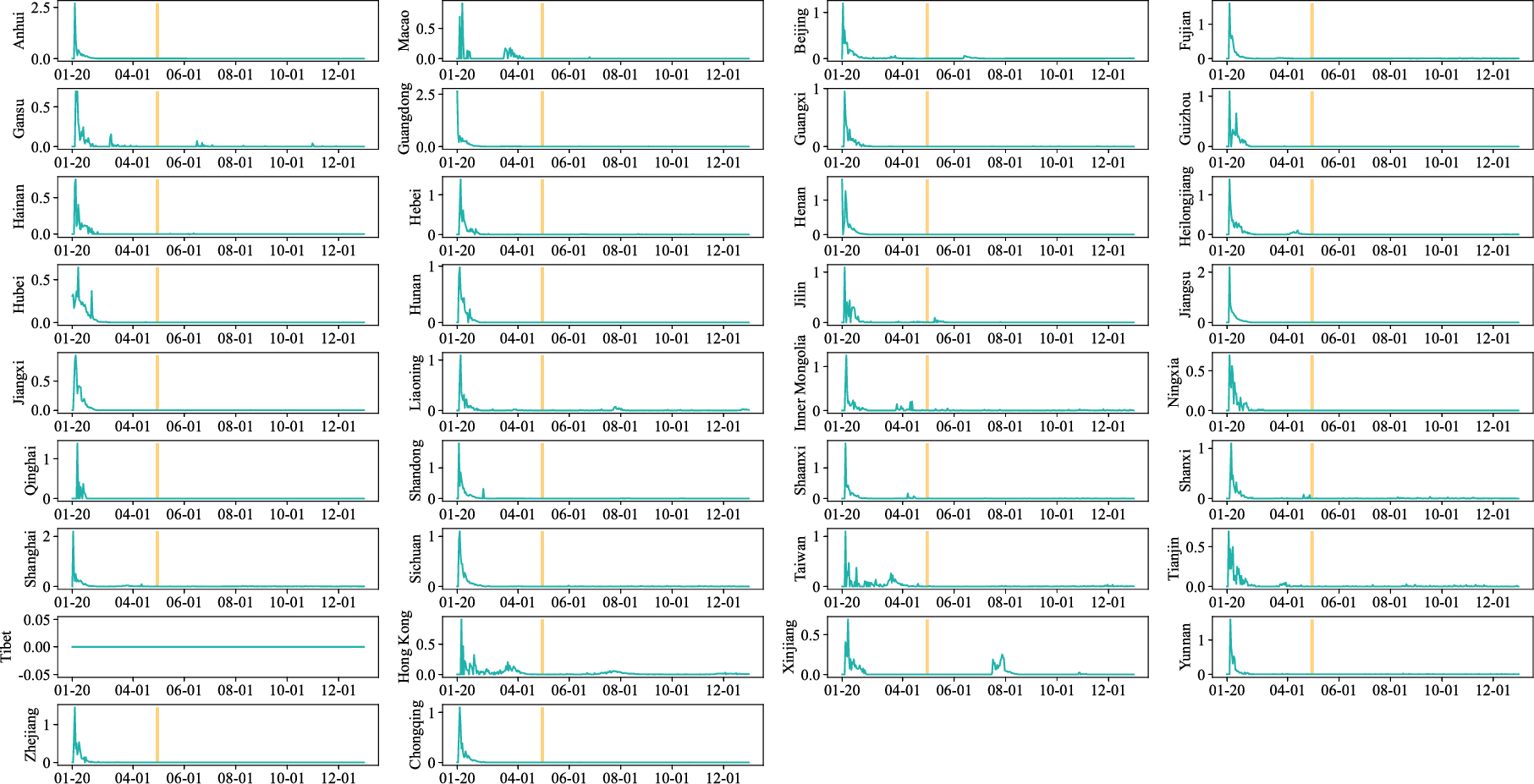

Supplement: S1 Fig — The green line is the growth rate of SARS-CoV-2 in each province, and the yellow line is April 30, 2020, which is used to select the time period. (TIF) [file pone.0285179.s001.tif]

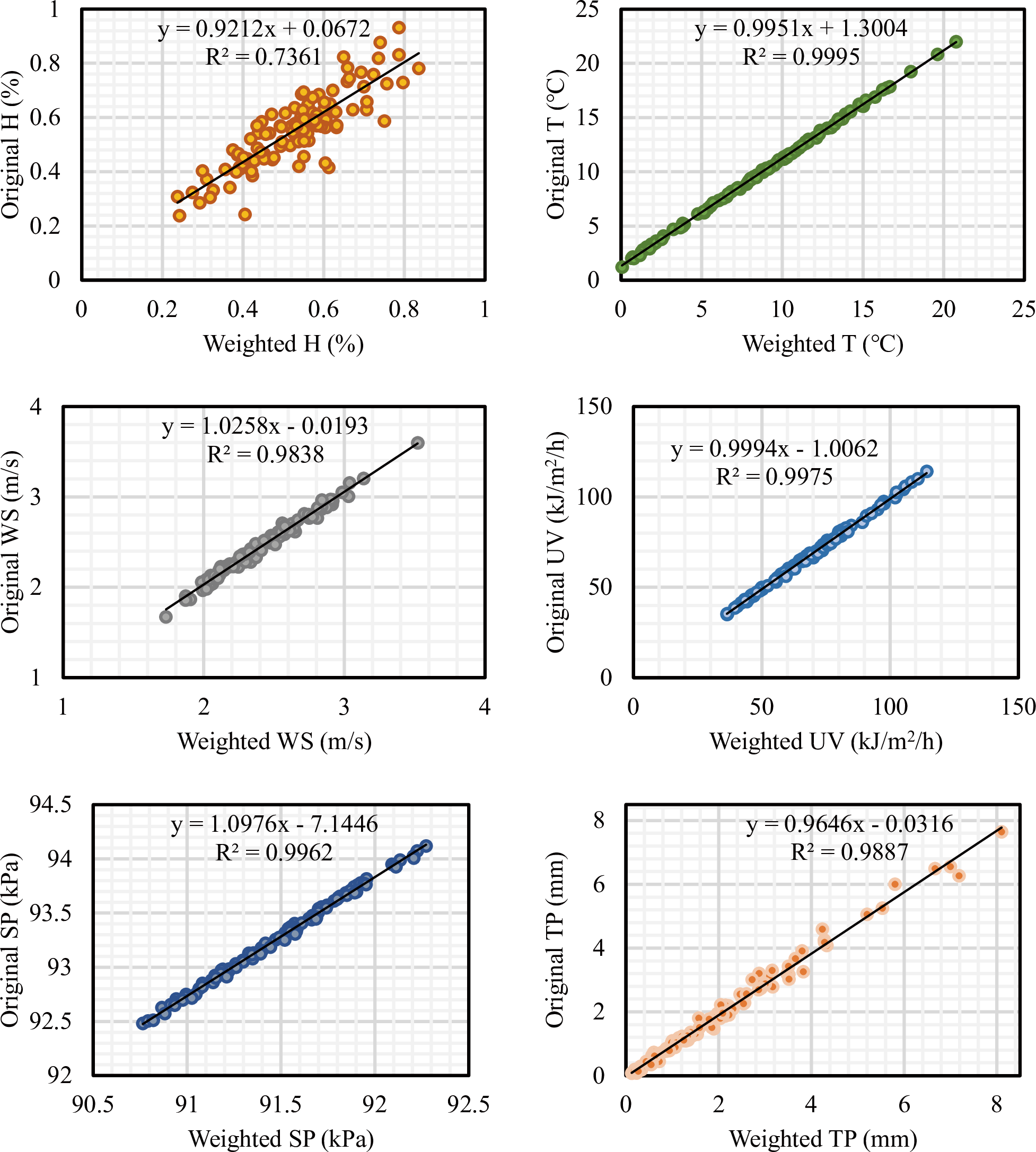

Supplement: S2 Fig — Notes: Original versus weighted data. Take Beijing and Guangdong for example. Abbreviations: growth rate of SARS-CoV-2 (GR), specific humidity (H), 2-meter temperature (T), wind speed (WS), ultraviolet (UV), surface pressure (SP), and total precipitation (TP). (TIF) [file pone.0285179.s002.tif]

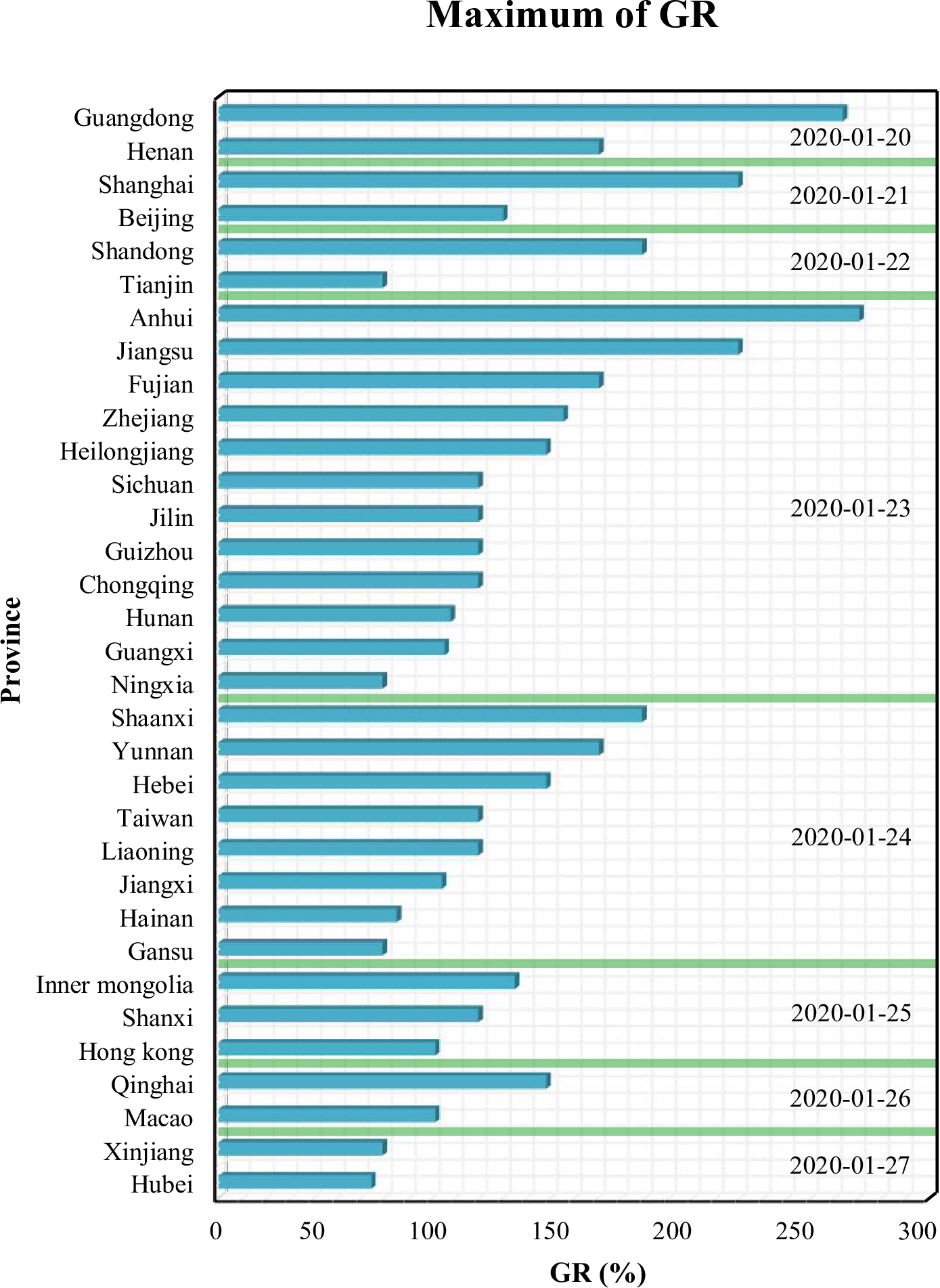

Supplement: S3 Fig — (TIF) [file pone.0285179.s003.tif]

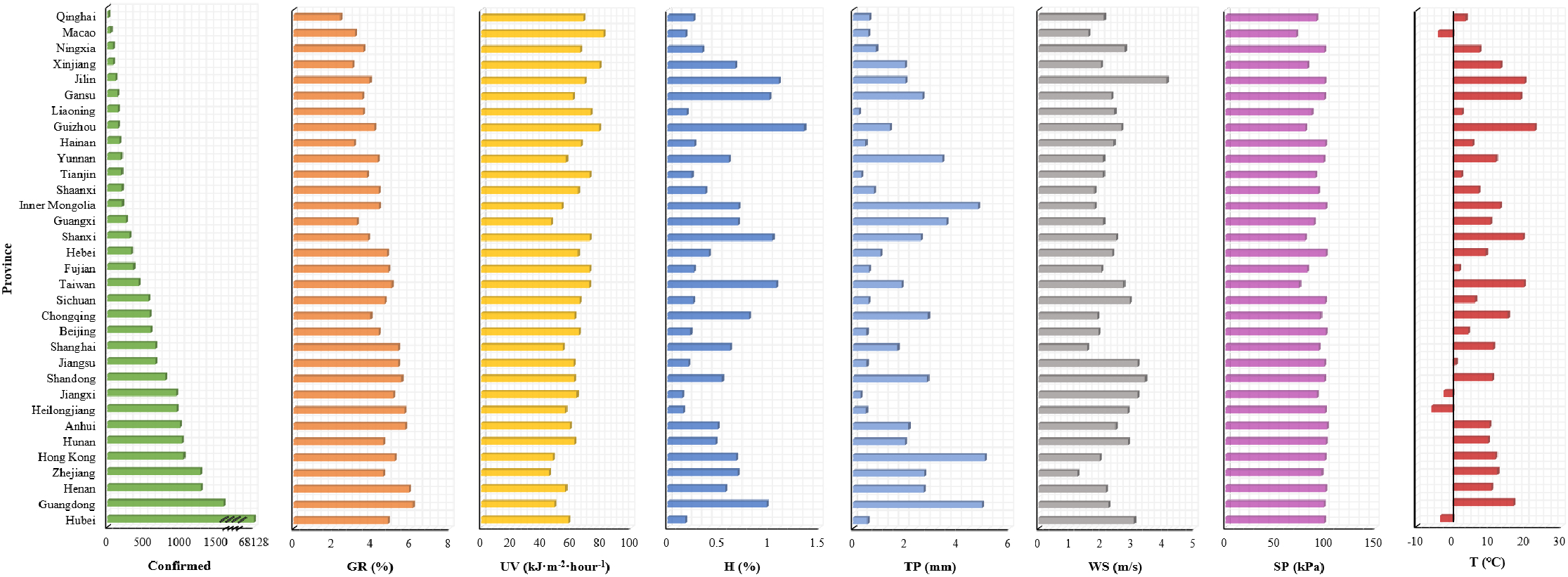

Supplement: S4 Fig — Notes: growth rate of SARS-CoV-2 (GR), specific humidity (H), 2-meter temperature (T), wind speed (WS), ultraviolet (UV), surface pressure (SP), and total precipitation (TP). (TIF) [file pone.0285179.s004.tif]

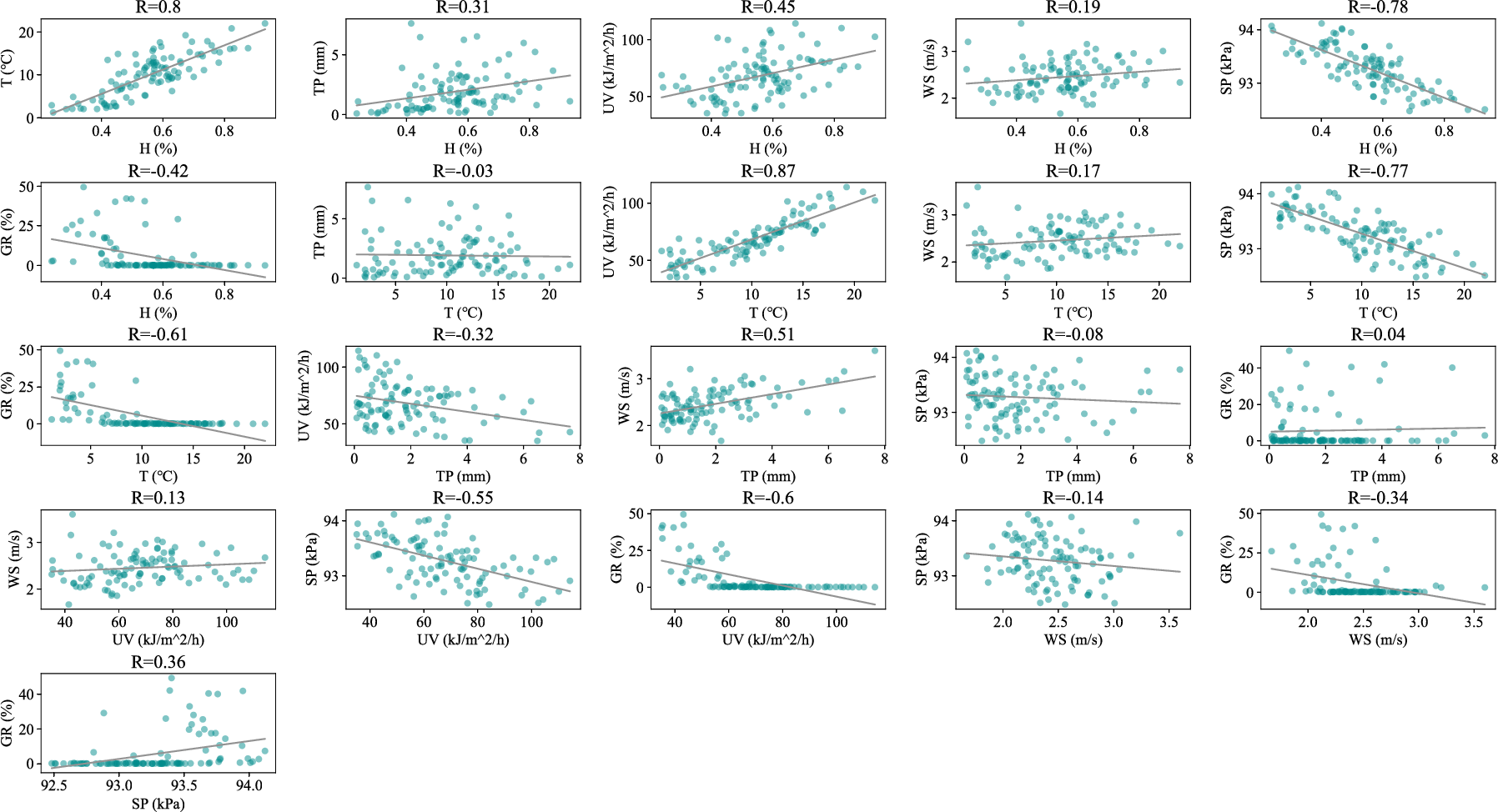

Supplement: S5 Fig — Notes: growth rate of SARS-CoV-2 (GR), specific humidity (H), 2-meter temperature (T), wind speed (WS), ultraviolet (UV), surface pressure (SP), and total precipitation (TP). (TIF) [file pone.0285179.s005.tif]

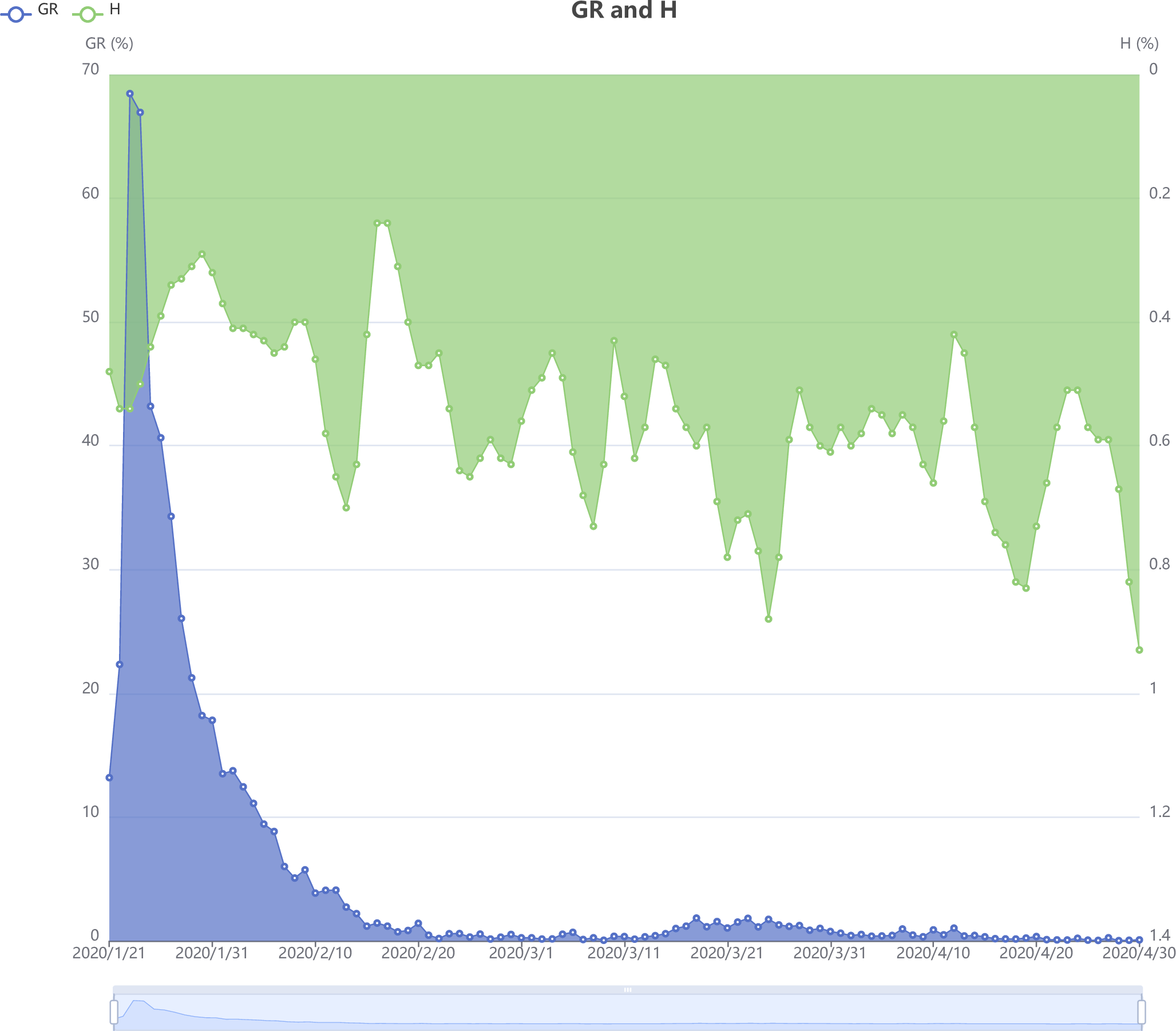

Supplement: S6 Fig — Notes: growth rate of SARS-CoV-2 (GR), specific humidity (H), 2-meter temperature (T), wind speed (WS), ultraviolet (UV), surface pressure (SP), and total precipitation (TP). (ZIP) [file pone.0285179.s006.zip › S6a_Fig.tif]

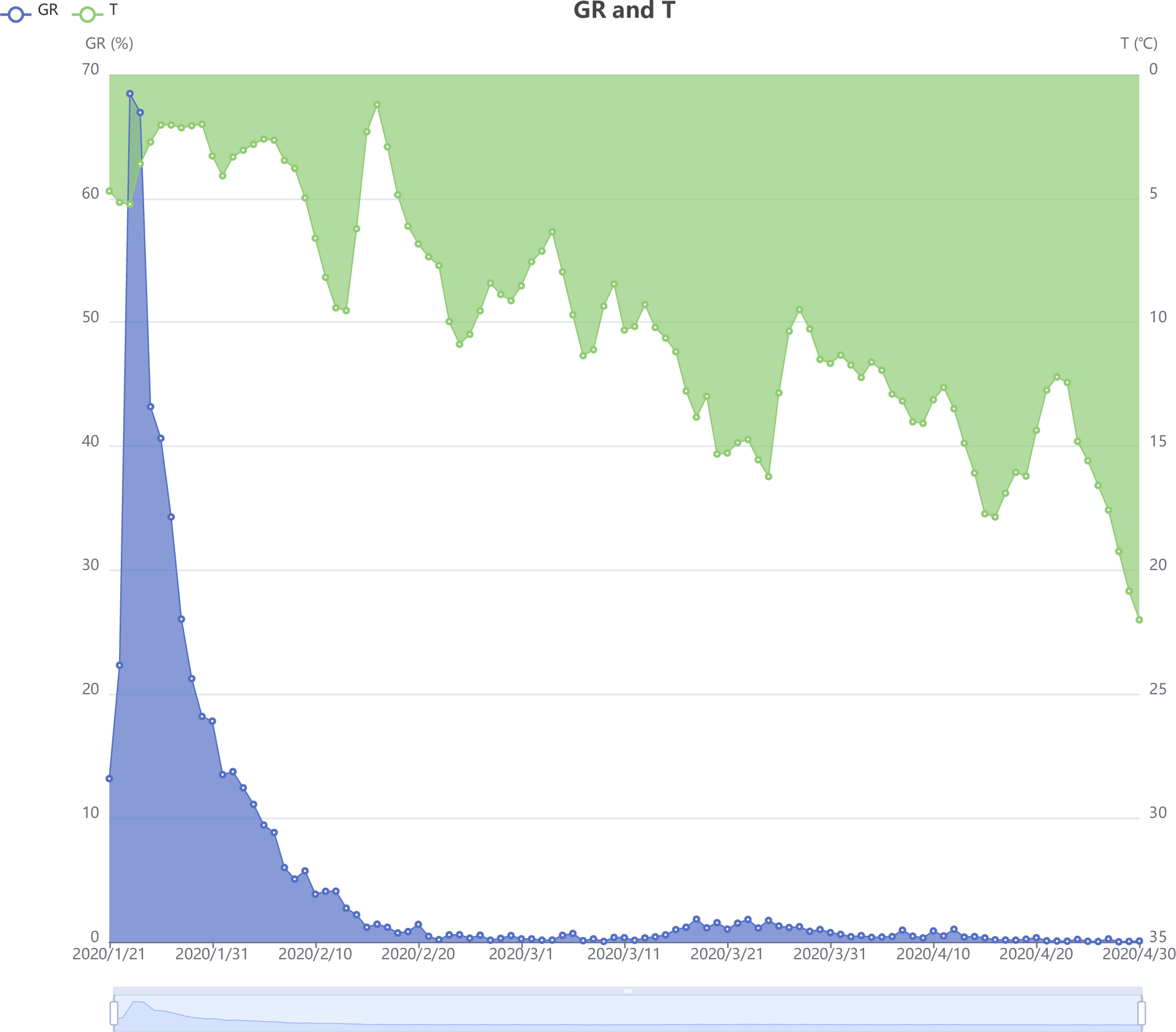

Supplement: S6 Fig — Notes: growth rate of SARS-CoV-2 (GR), specific humidity (H), 2-meter temperature (T), wind speed (WS), ultraviolet (UV), surface pressure (SP), and total precipitation (TP). (ZIP) [file pone.0285179.s006.zip › S6b_Fig.tif]

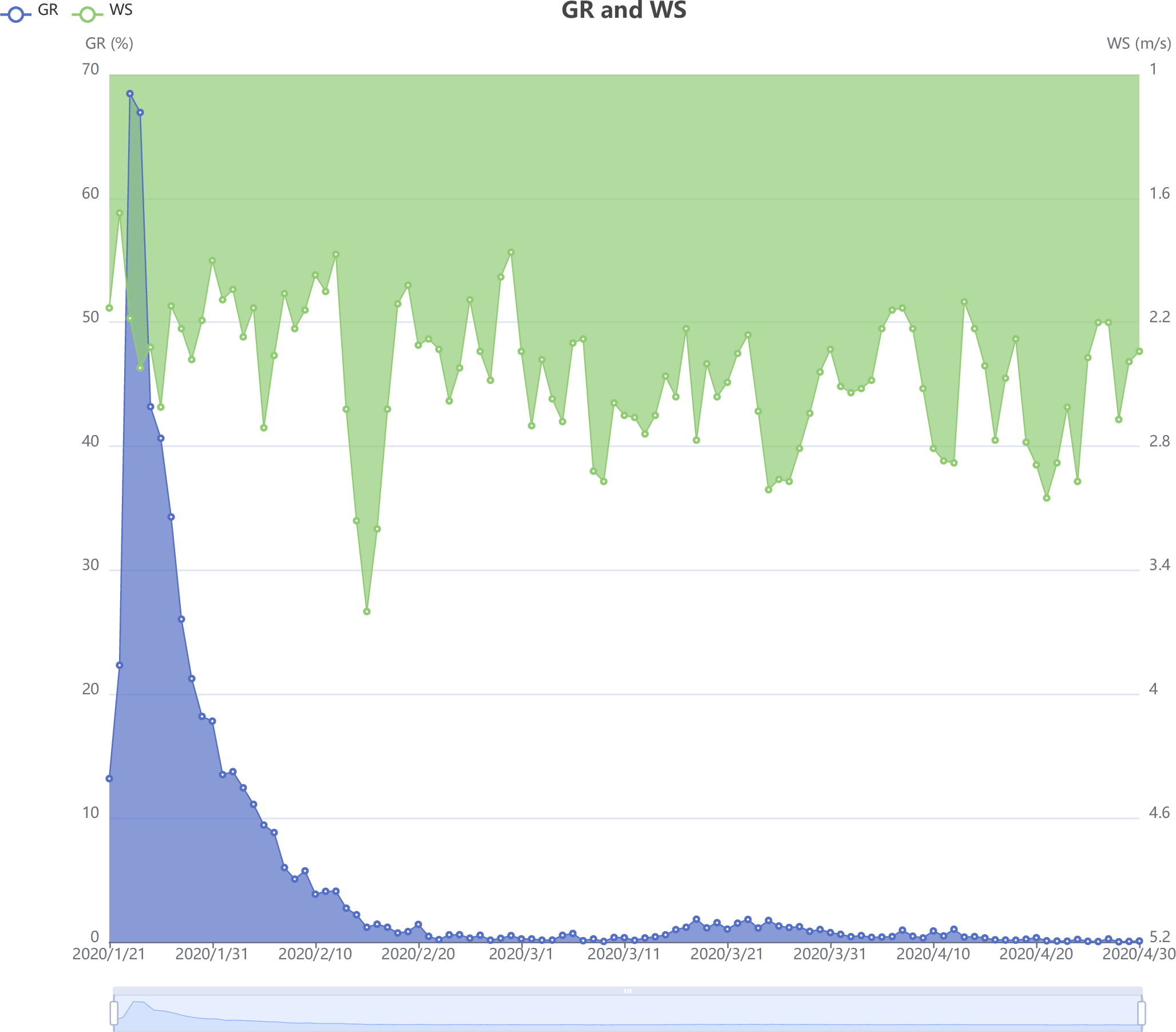

Supplement: S6 Fig — Notes: growth rate of SARS-CoV-2 (GR), specific humidity (H), 2-meter temperature (T), wind speed (WS), ultraviolet (UV), surface pressure (SP), and total precipitation (TP). (ZIP) [file pone.0285179.s006.zip › S6c_Fig.tif]

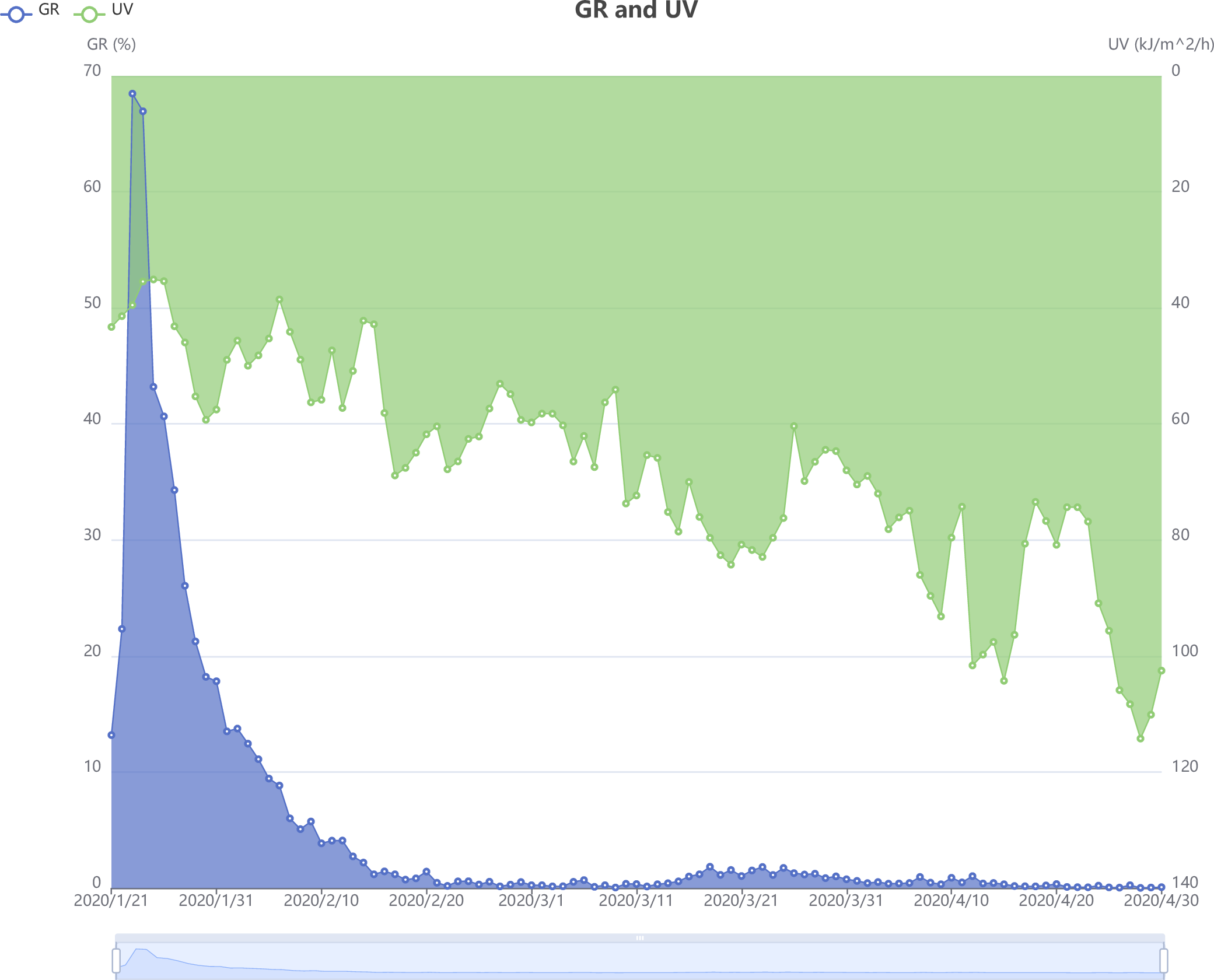

Supplement: S6 Fig — Notes: growth rate of SARS-CoV-2 (GR), specific humidity (H), 2-meter temperature (T), wind speed (WS), ultraviolet (UV), surface pressure (SP), and total precipitation (TP). (ZIP) [file pone.0285179.s006.zip › S6d_Fig.tif]

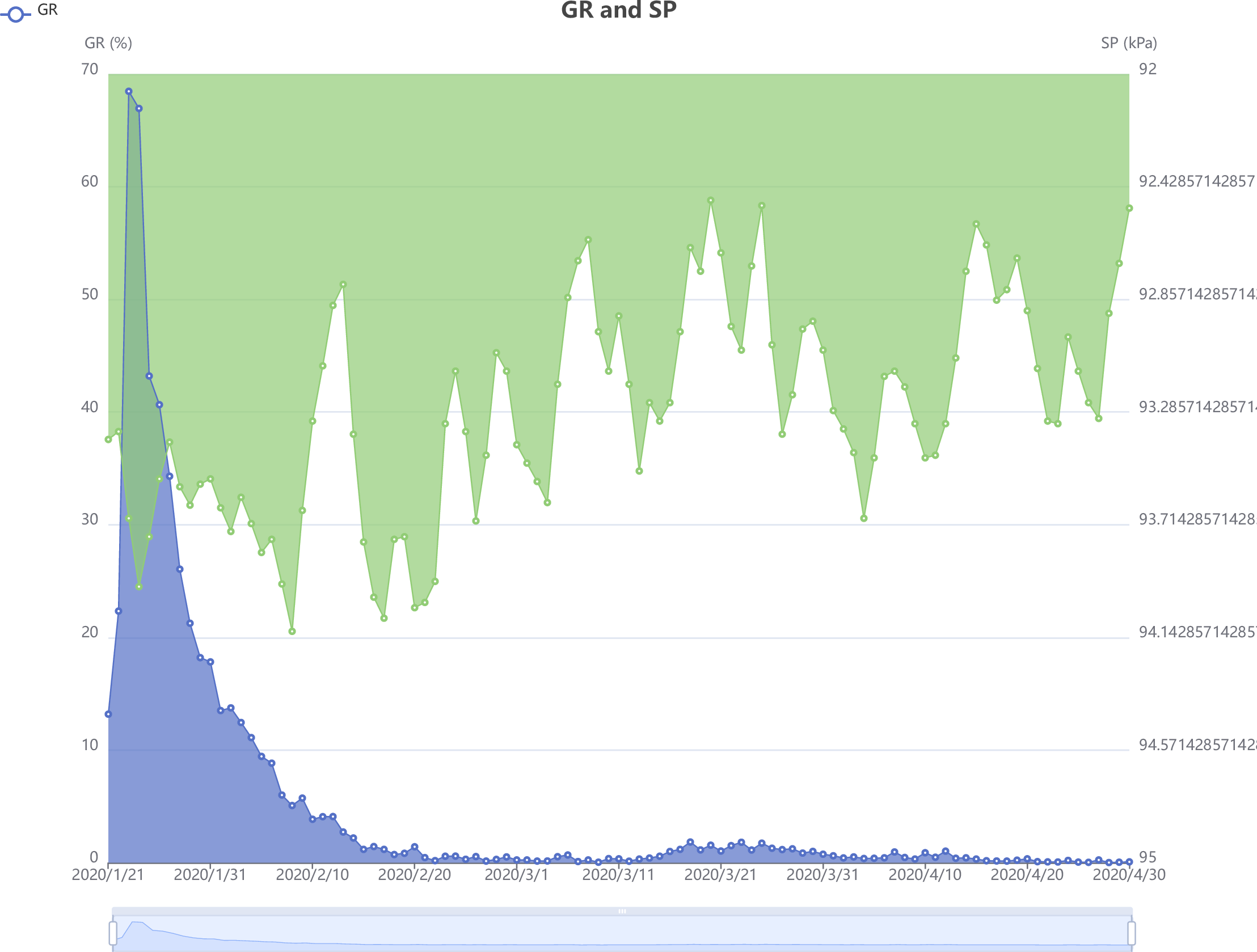

Supplement: S6 Fig — Notes: growth rate of SARS-CoV-2 (GR), specific humidity (H), 2-meter temperature (T), wind speed (WS), ultraviolet (UV), surface pressure (SP), and total precipitation (TP). (ZIP) [file pone.0285179.s006.zip › S6e_Fig.tif]

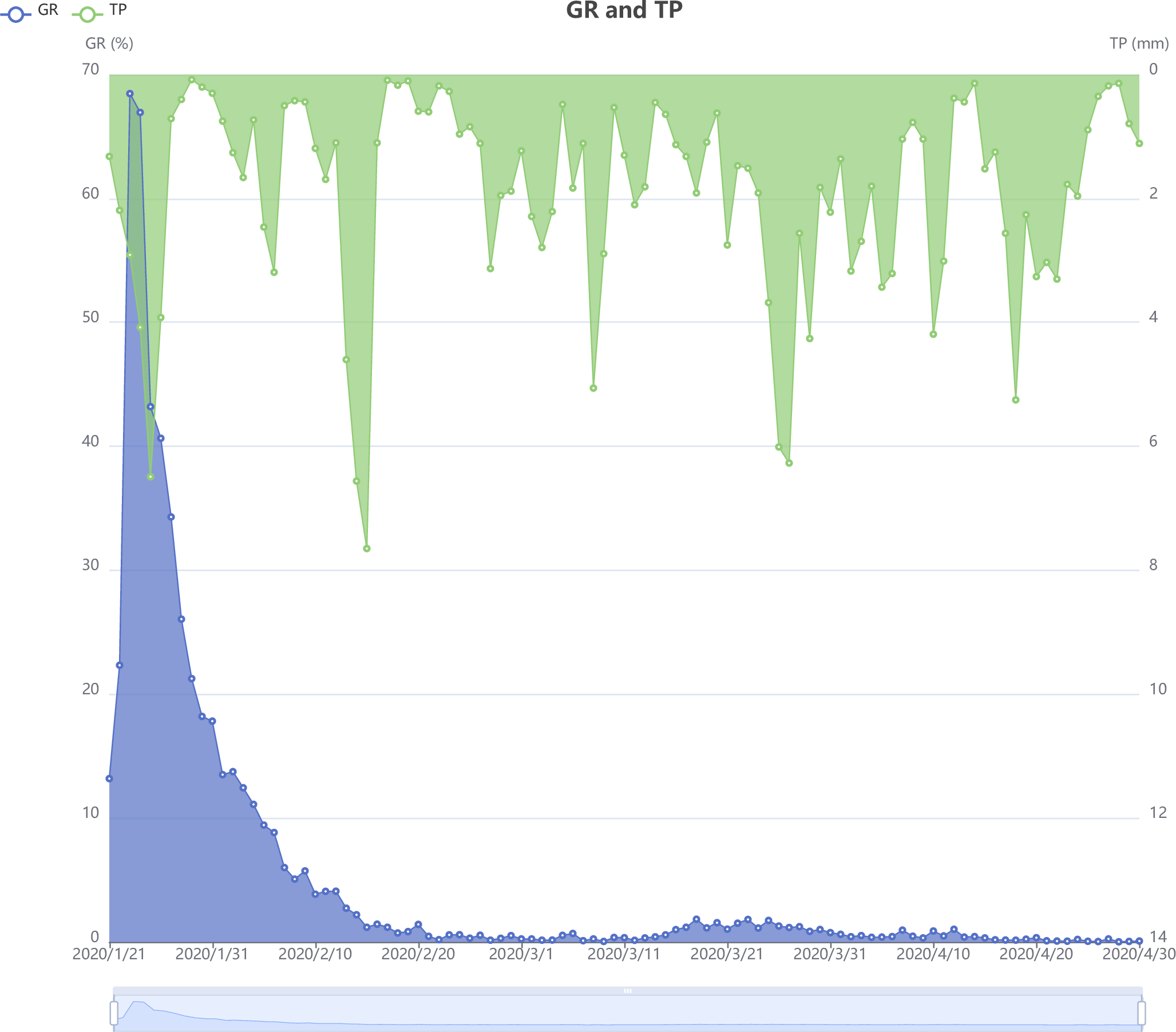

Supplement: S6 Fig — Notes: growth rate of SARS-CoV-2 (GR), specific humidity (H), 2-meter temperature (T), wind speed (WS), ultraviolet (UV), surface pressure (SP), and total precipitation (TP). (ZIP) [file pone.0285179.s006.zip › S6f_Fig.tif]

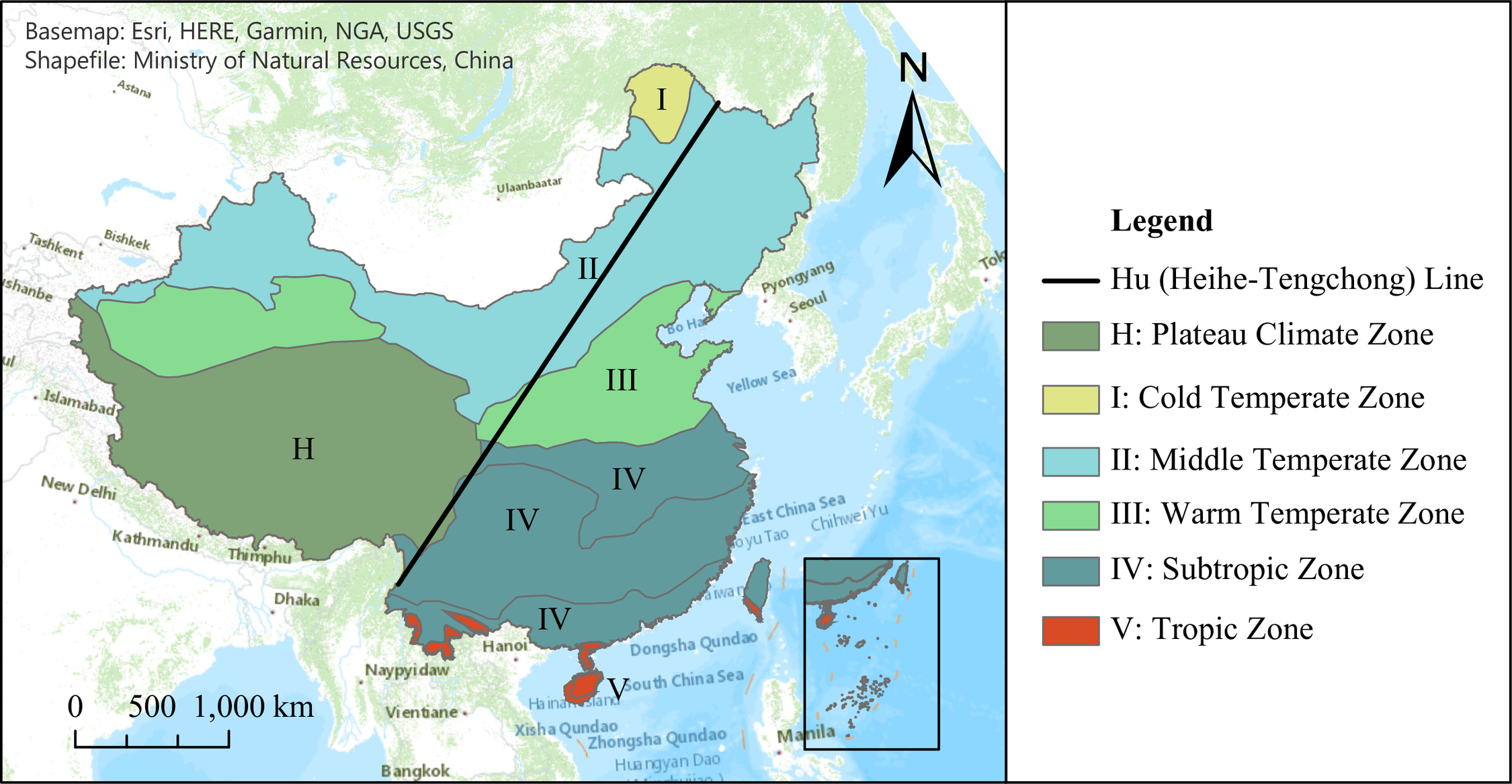

Supplement: S7 Fig — (TIF) [file pone.0285179.s007.tif]
