# Supplementary material for: Impact of climate change on SARS-CoV-2 epidemic in China
Source: PLoS One. 2023 Jul 27;18(7):e0285179. doi: 10.1371/journal.pone.0285179 (PMC10374073; doi:10.1371/journal.pone.0285179)
Supplement: S1 Table — Notes: growth rate of SARS-CoV-2 (GR), specific humidity (H), 2-meter temperature (T), wind speed (WS), ultraviolet (UV), surface pressure (SP), and total precipitation (TP). (DOCX) [file pone.0285179.s008.docx]

**S1 Table. Statistical information of each factor.**

|  | **GR** | **H** | **SP** | **T** | **TP** | **UV** | **WS** |
| --- | --- | --- | --- | --- | --- | --- | --- |
| **Min** | 0 | 0 | 0 | -5.85 | 0 | 0 | 0 |
| **Max** | 6.09 | 1.35 | 101.46 | 22.63 | 5.05 | 81.66 | 4.11 |
| **Q1** | 3.57 | 0.24 | 88.20 | 2.55 | 0.55 | 56.09 | 1.97 |
| **Median** | 4.38 | 0.48 | 98.03 | 9.86 | 1.56 | 63.01 | 2.29 |
| **Q3** | 5.08 | 0.70 | 99.34 | 13.00 | 2.71 | 69.14 | 2.77 |
| **IQR** | 1.51 | 0.46 | 11.14 | 10.45 | 2.16 | 13.05 | 0.80 |
| **Mean** | 4.28 | 0.52 | 90.53 | 8.57 | 1.76 | 61.33 | 2.32 |
| **S.D.** | 1.19 | 0.34 | 18.05 | 7.44 | 1.43 | 14.14 | 0.72 |

Notes: growth rate of SARS-CoV-2 (GR), specific humidity (H), 2-meter temperature (T), wind speed (WS), ultraviolet (UV), surface pressure (SP), and total precipitation (TP).
