# Supplementary material for: Impact of climate change on SARS-CoV-2 epidemic in China
Source: PLoS One. 2023 Jul 27;18(7):e0285179. doi: 10.1371/journal.pone.0285179 (PMC10374073; doi:10.1371/journal.pone.0285179)
Supplement: S2 Table — Notes: * represents P-value < 0.05, and · represents P-value < 0.01. Abbreviations: growth rate of SARS-CoV-2 (GR), specific humidity (H), 2-meter temperature (T), wind speed (WS), ultraviolet (UV), surface pressure (SP), and total precipitation (TP). (DOCX) [file pone.0285179.s009.docx]

**S2 Table. Correlation of various factors.**

|  | **GR** | | **UV** | | **H** | | **T** | | **TP** | | **WS** | | **SP** | |
| --- | --- | --- | --- | --- | --- | --- | --- | --- | --- | --- | --- | --- | --- | --- |
| **GR** | 1 |  |  |  |  |  |  |  |  |  |  |  |  |  |
| **UV** | -0.53 | * | 1 |  |  |  |  |  |  |  |  |  |  |  |
| **H** | -0.33 | * | 0.45 | * | 1 |  |  |  |  |  |  |  |  |  |
| **T** | -0.51 | * | 0.87 | * | 0.80 | * | 1 |  |  |  |  |  |  |  |
| **TP** | 0.14 |  | -0.32 | * | 0.31 | * | -0.03 |  | 1 |  |  |  |  |  |
| **WS** | -0.23 | * | 0.13 |  | 0.19 | · | 0.17 | · | 0.51 | * | 1 |  |  |  |
| **SP** | 0.35 | * | -0.55 | * | -0.78 | * | -0.77 | * | -0.08 | * | -0.14 | * | 1 |  |

Notes: * represents *P-value* < 0.05, and · represents *P-value* < 0.01. Abbreviations: growth rate of SARS-CoV-2 (GR), specific humidity (H), 2-meter temperature (T), wind speed (WS), ultraviolet (UV), surface pressure (SP), and total precipitation (TP).
