# Supplementary material for: Impact of climate change on SARS-CoV-2 epidemic in China
Source: PLoS One. 2023 Jul 27;18(7):e0285179. doi: 10.1371/journal.pone.0285179 (PMC10374073; doi:10.1371/journal.pone.0285179)
Supplement: S3 Table — Notes: R-squared = 0.85, Adjusted R-squared = 0.81, and Prob(F-statistic) < 0.001. Abbreviations: growth rate of SARS-CoV-2 (GR), specific humidity (H), 2-meter temperature (T), wind speed (WS), ultraviolet (UV), surface pressure (SP), and total precipitation (TP). (DOCX) [file pone.0285179.s010.docx]

**S3 Table. Coefficient of cumulative effect analysis.**

| **Lags** | **Coefficient** | | | | | | |
| --- | --- | --- | --- | --- | --- | --- | --- |
|  | **H** | **WS** | **SP** | **T** | **UV** | **TP** | **CONSTANT** |
| **0** | 1.83 | -1.66 | 0.98 | -0.47 | -0.05 | 4.12E-3 | 1,447.00 |
| **1** | 0.32 | -1.72 | 1.12 | -0.35 | -0.03 | -0.02 | - |
| **2** | -0.91 | -1.75 | 1.23 | -0.24 | -0.03 | -0.04 | - |
| **3** | -1.86 | -1.74 | 1.31 | -0.16 | -0.02 | -0.04 | - |
| **4** | -2.54 | -1.70 | 1.37 | -0.09 | -0.01 | -0.03 | - |
| **5** | -2.93 | -1.63 | 1.40 | -0.04 | -0.01 | -0.01 | - |
| **6** | -3.05 | -1.52 | 1.41 | -0.01 | -0.01 | 0.02 | - |
| **7** | -2.89 | -1.38 | 1.39 | 3.21E-03 | -0.01 | 0.07 | - |
| **8** | -2.46 | -1.21 | 1.34 | -1.86E-03 | -0.02 | 0.12 | - |
| **9** | -1.74 | -1.01 | 1.27 | -0.03 | -0.03 | 0.19 | - |
| **10** | -0.75 | -0.78 | 1.17 | -0.07 | -0.04 | 0.27 | - |
| **11** | 0.52 | -0.51 | 1.04 | -0.13 | -0.05 | 0.36 | - |
| **Sum** | -16.47 | -16.61 | 15.02 | -1.58 | -0.30 | 0.90 | - |

Notes: R-squared = 0.85, Adjusted R-squared = 0.81, and Prob (F-statistic) < 0.001. Abbreviations: growth rate of SARS-CoV-2 (GR), specific humidity (H), 2-meter temperature (T), wind speed (WS), ultraviolet (UV), surface pressure (SP), and total precipitation (TP).
